# Progresses, Challenges, and Prospects of Genome Editing in Soybean (*Glycine max*)

**DOI:** 10.3389/fpls.2020.571138

**Published:** 2020-10-22

**Authors:** Hu Xu, Lixiao Zhang, Kang Zhang, Yidong Ran

**Affiliations:** Tianjin Genovo Biotechnology Co., Ltd., Tianjin, China

**Keywords:** soybean, genome editing, CRISPR, ZFNs, TALENs, crop improvement

## Abstract

Soybean is grown worldwide for oil and protein source as food, feed and industrial raw material for biofuel. Steady increase in soybean production in the past century mainly attributes to genetic mediation including hybridization, mutagenesis and transgenesis. However, genetic resource limitation and intricate social issues in use of transgenic technology impede soybean improvement to meet rapid increases in global demand for soybean products. New approaches in genomics and development of site-specific nucleases (SSNs) based genome editing technologies have expanded soybean genetic variations in its germplasm and have potential to make precise modification of genes controlling the important agronomic traits in an elite background. ZFNs, TALENS and CRISPR/Cas9 have been adapted in soybean improvement for targeted deletions, additions, replacements and corrections in the genome. The availability of reference genome assembly and genomic resources increases feasibility in using current genome editing technologies and their new development. This review summarizes the status of genome editing in soybean improvement and future directions in this field.

## Introduction

Soybean [*Glycine max* (L.) Merr.] is becoming an important agricultural commodity and grown worldwide for feed and food products. It is one of major protein source for human nutrition as food, as well as feed for livestock and fish since soybean seed contains about 40% protein and about 20% oil (Singh, 2017). Recently, soybean is also used as a source of biofuel. Soybean root and a rhizobacterium, *Bradyrhizobia japonicum*, can normally establish rhizobia-legume symbiosis which fixes nitrogen and improves soil quality. The United States, Brazil, and Argentina produced more than 80% of global soybean annually. China and India are the two major soybean growing countries in Asia^1^. Soybean has been one of the fastest growing major crops for several decades and its production is boosted recently by increasing demand from China. About 30% of world’s production is consumed in China which is becoming the largest soybean importer in the world (Hart, 2017). Therefore, the global soybean market is driven by two major producers (United States and Brazil) and one major consumer (China) (Gale et al., 2019).

Taxonomy of the genus Glycine is classified and well characterized using morphological evaluation, cytogenetic analysis and molecular phylogenetics (Chung and Singh, 2008). The genus includes two subgenera, one of which contains cultivated soybean (*G. max*) and its wild relative (*G. soya*), both of which are annual Asian species and are descendants from an ancient genome duplication events (Shoemaker et al., 2006). Therefore, soybean is classified as a paleopolyploid and has 40 chromosomes (2n = 40) (Karpechenko, 1925), which are small size (1.42–2.84 lm) with similar and distinguishing morphology (Sen and Vidyabhusan, 1960). Twenty molecular linkage groups (MLGs) have been developed using primarily restriction fragment length polymorphism (RFLP), amplified fragment length polymorphism (AFLP) and simple sequence repeat (SSR) loci (Xia et al., 2007) and single nucleotide polymorphisms (SNPs) (Choi et al., 2007; Akond et al., 2013). Soybean has very limited genetic diversity since most cultivars are found to be selected from the original same group of progenitors (Singh, 2017). The limitation of genetic resource is the major challenge for soybean improvement to overcome the significant constraints for farming and production caused by climate changing, reduced agricultural land availability and increased biotic and abiotic stresses. Therefore, improved molecular-based breeding and genetic engineering technologies are necessary to break through the bottleneck for further improvement of soybean agronomical traits and to guarantee yield increases for satisfying future demands of soybean in global market to feed nearly 10 billion people by 2050. Except for introducing genetic source from wild relatives, scientists have continuously worked to modify soybean genome using molecular genetics and genomics approaches. Transgenesis based biotechnologies has extensively been used in soybean to improve its agronomic traits. For the past four decades, transgenesis have been used to understand basic plant biology and can break the bottleneck of reproductive isolation, which transfers exogenous genes into elite variety background to generates novelty traits. They have been used for soybean improvement and made soybean to be one of the major transgenic crops grown commercially in the world. However, like other transgenic crops, the random integration of transgenes into the host genome and multiple copies can cause unstable and off-target effects, which also cause public concern for human consumption, and commercialization of soybean as genetically modified crop is restricted by tedious and costly regulatory evaluation processes.

Mutagenesis is another way to expand soybean germplasm. Conventionally, soybean gene can be mutated using random mutagens including radiation such as X-rays, fast neutrons, and gamma rays, chemicals such as EMS (ethyl methanesulfonate) and NMU (N-nitroso-N methylurea), and biological mutagenesis such as T-DNA insertion and transposons (Liu et al., 2017; O’Rourke et al., 2017). Random mutagenesis is heritable and stable but requires intensive screening and specific techniques such as targeting induced local lesions in genomes (TILLING) to identify mutant phenotypes. Such techniques are time consuming and can be expensive (Liu et al., 2017). In most cases, it is impossible to obtain specific alleles known to confer certain phenotypes due to imprecise mutation. In the last 2 decades, site-directed nucleases (SDNs) or site-specific nucleases (SSNs) based new biotechnologies such as Zinc Finger Nucleases (ZFNs), Transcription Activator-Like Effector Nucleases (TALENs) or the more recent Clustered Regularly Interspaced Short Palindromic Repeat (CRISPR), has been developed for mutagenesis. As very useful tools, multiple SDN platforms have been integrated into the plant breeding programmers (Chen K. et al., 2019; Zhang Y. et al., 2019) including soybean. SDNs have been developed for genome editing (GE) and have induced mutations with unprecedented precision, which includes all type mutations existed during crop evolution processes including domestication and breeding. Hence, novel genome editing technologies are expected to accelerate the speed of breeding programs as the main option for revealing gene function and producing new varieties. In this review, we will summarize the status of soybean genome editing, address current bottleneck and discuss future perspectives in this field.

## Genome Editing Tool Development and Availability for Plant Genome Editing

**FIGURE 1:**
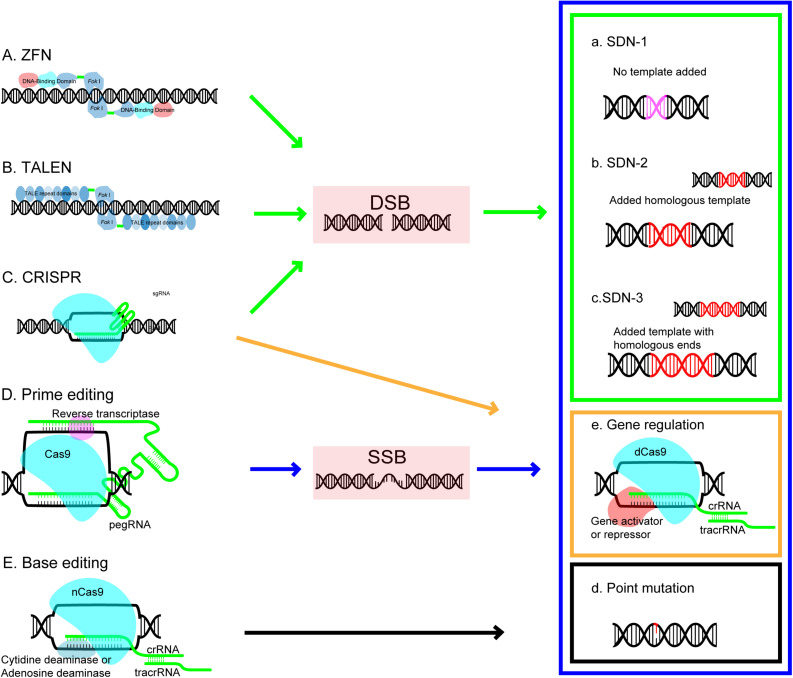
Genome editing platforms and editing outcomes. Each editing platform (arrow) and its outcomes (rectangular) are coded with the same color. ZFN, zinc-finger nuclease; TALEN, transcription activator-like effector nuclease; CRISPR, clustered regulatory interspaced short palindromic repeat; DSB, double strand breaks; SSB, single strand breaks; Outcomes of GE created by site-directed nucleases (SDN) includes: SDN1-the approach involves DNA breaks repair through DNA repair mechanisms in the host cellular without using an added repair template; SDN2-the approach involves the break repair *via* HR using an added homologous repair template; and SDN3-the approach involves DNA break repair *via* either HDR or NHEJ pathway using an added DNA template containing nonhomologous sequences but with homologous ends.

The basic concept of SDNs based genome editing is that nucleases can be designed to recognize the desired target site in DNA and induce a cleavage which make a double stranded break (DSB), then the DSB can be naturally repaired by the DNA own repair mechanism in cell either by endogenous repair pathways through non-homologous end-joining (NHEJ) or through homologous-directed repair pathways (HDR) (Ran et al., 2017; Figure 1). As illustrated in Figure 1, the NHEJ repair is the error prone pathway and possible to induce random insertions and deletions which disrupt the reading frame and lead to targeted gene knockouts; the HDR pathway, a precise exchange of homologous sequence involved process using an externally added homologous DNA repair template, results in gene replacement or targeted insertion (Voytas and Gao, 2014; Bortesi and Fischer, 2015). GE technology is becoming increasingly diversified and sophisticated (Chen F. et al., 2019). Based on genome editing difference processes occur during repair DNA breaks, the basic outcomes of genome editing can be divided into three categories (Sprink et al., 2016). SDN1 (the approach involves DNA breaks repair through DNA repair mechanisms in the host cellular without using an added repair template), SDN2 (the approach involves the break repair *via* HR using an added homologous repair template), and SDN3 (the approach involves DNA break repair *via* either HDR or NHEJ pathway using an added DNA template containing non-homologous sequences but with homologous ends). Emerging of new technology makes base editing and transcriptional regulation of target gene as additional outcomes (Figure 1).

Zinc Finger Nucleases and TALENs are earlier GE platforms generations and each customized ZFN or TALEN protein needs to be genetically manufactured to generate DSBs at the targeting location, and the GE using these platforms has been demonstrated in many plants (Joung and Sander, 2013). However, some drawbacks of these platforms has limited their applications, which include the difficulty to engineer ZFNs and TALENs due to the highly repetitive sequences and complex nature of the interaction between ZFN and DNA (Bortesi and Fischer, 2015), and the complication to make them due to minimal requirement of a pair of ZFNs or TALENs for both the up-stream and the down-stream regions of the targeting site (Beumer et al., 2013). Many ZFNs or TALENs would be required to achieve multiplexing which edit several targets simultaneously. Since zinc finger nucleases were used in tobacco in Wright et al. (2005), various GE technologies have gradually been adapted in plant along with their development, such as TALENs which editing activity was confirmed in plant (Cermak et al., 2011). However, the application of GE in plant mutagenesis and trait improvement using ZFNs and TALENs have been restricted due to the technique limitation of the ZFNs and TALENs (Petolino, 2015; Weeks et al., 2016). The CRISPR/Cas system, a newly developed GE platform (Doudna and Charpentier, 2014), comprises Cas proteins and a single guide RNA (sgRNA) with a hairpin structure targeting a 20-base pair (bp) DNA sequence site (Figure 1). Based on phylogenetic, structural and functional characteristics of their Cas genes and the nature of the interference complex, CRISPR/Cas systems have been classified into class1 and class 2 systems. Class 1 systems involve in multi-Cas protein complexes for interference and are further divided into type I, III and IV, whereas Class 2 systems involve in interference with single effector proteins in the pre-CRISPR RNA (pre-crRNA) processing and is composed of subclass type II, V, and VI, which include Cas9 (type II), Cas12a-e (type V) and Cas13a-d (type VI) (Makarova et al., 2015). The type II CRISPR/Cas9 system, which is based on RNA-guided interference with DNA, has been adapted for genome editing (Koonin et al., 2017) and has been the first system confirmed to cleave DNA *in vitro* and in eukaryotic cells (Jinek et al., 2012; Gilbert et al., 2013; O’Connell et al., 2014). It is a revolutionized mutagenesis system due to its easy design, flexible and easy operation, robust activity and cost saving property (Doudna and Charpentier, 2014; Murugan et al., 2017). Cas9 is required to assemble with the single guide RNA, the complex then recognize and bind to the targeting DNA sequences with a protospacer adjacent motif (PAM), finally the Cas9 nuclease induces a DSB in the 20 bp targeted DNA sequence adjacent to the PAM. SpCas9 (*Streptococcus pyogenes* Cas9) based CRISPR/Cas9, which recognizes a PAM (NGG) (N means any nucleotide), is the most commonly used GE system. CRISPR system has been evolved during the last decade. Recent advance of CRISPR technology includes:

### The Expanding GE Toolbox Comprises Precise Cas9 Variants and Orthologues, Wider Genome Accessibility by Recognizing a Simpler PAM

Cas9 enzymes originated from other bacteria have been discovered and nearly 10 Cas9 orthologues have been evaluated and developed as tools for genome editing, which includes *Staphylococcus aureus* (SaCas9), *Campylobacter jejuni* Cas9 (CjCas9), and others (Table 1). Natural Cas9 proteins identified can be modified to recognize different PAMs and engineered Cas9 with various PAMs is available for GE such as EQR-Cas9 (NGAG PAM), SaKKH-Cas9 (NNNRRT), SpCas9-NG (NG), VQR-Cas9 (NGA), and others (Table 1). Many of these SpCas9 variants have been used in GE for plants (summarized in Zhang Y. et al., 2019).

**TABLE 1:** Orthologous and variants of Cas9 and Cas enzymes.

Cas enzyme	Source	Subtype	PAM*	Cas enzyme	Source	Subtype	PAM*
SaCas9KKH	*Staphylococcus aureus*	Type II	NNNRRT	AsCpf1-RR	*Acidaminococcu s*sp.	Type V-A	TYCV
St1Cas9	*Streptococcus thermophiles*	Type II	NNAGAAW	AsCpf1-RVR	*Acidaminococcu s*sp.	Type V-A	TATV
St3Cas9	*Streptococcus thermophiles*	Type II	NGGNG	LbCpf1-RR	*Lachnospiraceae bacterium*	Type V-A	CCCC and TYCV
ScCas9	*Streptococcus canis*	Type II	NNG	LbCpf1-RVR	*Lachnospiraceae bacterium*	Type V-A	TATV
CjCas9	*Campylobacter jejuni*	Type II	NNNNRYAC	FnCpf1-RR	*Francisella novicida*	Type V-A	CCCC and TYCV
FnCas9	*Francisella novicida*	Type II	NGG	FnCpf1-RVR	*Francisella novicida*	Type V-A	TATV
RHACas9	*Francisella novicida*	Type II	YG	Mb3Cpf1	*Moraxella bovoculi* AAX11_00205	Type V-A	RTTV
NmCas9	*Neisseria meningitides*	Type II	NNNNGATT	BsCpf1	Butyrivibrio sp. NC3005	Type V-A	NTTV
TdCas9	*Treponema denticola*	Type II	NAAAAN	TsCpf1	*Thiomicrospira* sp. XS5	Type V-A	NTTV
SpCas9	*Streptococcus pyogenes*	Type II	NGG	SmCms1	*Smithella* sp.	Type V-A	TTN
VQR-Cas9	*Streptococcus pyogenes*	Type II	NGA	MiCms1	*Microgenomates*	Type V-A	TTN
EQR-Cas9,	*Streptococcus pyogenes*	Type II	NGAG	ObCms1	Omnitrophica bacterium	Type V-A	TTN
VRERCas9	*Streptococcus pyogenes*	Type II	NGCG	SuCms1	Sulfuricurvum sp. PC08-66	Type V-A	TTN
SpCas9-NG	*Streptococcus pyogenes*	Type II	NG	AaCas12b (C2c1	*Alicyclobacillus acidiphilus*	Type V-B	TTN
xCas9	*Streptococcus pyogenes*	Type II	NG, GAA, GTA	DpbCasX (Cas12c)	*Deltaproteobacteria*	Type V-C	TTCN
QQR1-Cas9	*Streptococcus pyogenes*	Type II	NAAG	PlmCasX (Cas12c)	*Planctomycete*	Type V-C	TTCN
SaKKH-Cas9	*Staphylococcus aureus*	Type II	NNNRRT	VobCasY(Cas12d)	*Vogelbacteria*	Type V-D	TA
CPF1(Cas 12a)	*Prevotella* and *Francisella* 1	Type V-A	TTTC	KabCasY (Cas12d)	*Katanobacteria*	Type V-D	TA
FnCpf1	*Francisella novicida*	Type V-A	TTN	Cas13a (C2c2)	Leptotrichia shahii (LshCas13a)	Type VI	PFS: H
AsCpf1	*Acidaminococcu s*sp.	Type V-A	TTTV	LwaCas13a	Leptotrichia wadei	Type VI	Without PFS
LbCpf1	*Lachnospiraceae bacterium*	Type V-A	TTTV	PspCas13b	*Prevotella* sp. P5-125	Type VI	Without PFS

* (V = A, C, or G); R = A or G; Y = C or T; N = any of nucleotides (A, C, G, T); H = A, C, or U; PFS, a protospacer flanking site.

### The Discovery of Various Cas Enzymes With Unique PAMs and Engineering of CRISPR/Cas Components for Improved GE

Recently, a new class 2 type V-A Cas enzyme Cpf1 (formally known as Cas12a) from *Prevotella* and *Francisella* 1 was identified in the type II CRISPR systems and functionally characterized (Zetsche et al., 2015). Unlike Cas9 which prefer G-rich PAM, Cpf1 recognizes a T-rich region in target DNA sequence and induces a DSB with sticky-ends with a 5-nucleotide 5′ overhang downstream from the PAM (TTTC) site, which is able to make DNA DSB continuously and may result in insertion mutation through NHEJ pathway. Cpf1 owns both DNAase and RNase activity, which allows process a CRISPR array for multiplex. Cpf1 does not need tracrRNAs for crRNA biogenesis and it also has a shorter guide crRNA with about 43 bp compared to ∼80 bp of Cas9 sgRNA which lead easy synthesis and engineering of crRNA (Zetsche et al., 2015). Cpf1 variants such as FnCpf1 from *F. novicida*, AsCpf1 from *Acidaminococcu ssp*., and LbCpf1 from *Lachnospiraceae bacterium*, have been used in genome editing for many plant species (Zhang Y. et al., 2019). To broaden the target ranges for Cpf1 to recognizes PAMs different from the TTTV identified initially, modified variants with different PAM recognition have been generated and used for GE, such as AsCpf1-RR (TYCV PAM), and -RVR (TATV) (Gao et al., 2017); LbCpf1-RR (CCCC and TYCV) and LbCpf1-RVR (TATV) (Li et al., 2018); FnCpf1-RR (CCCC and TYCV) and FnCpf1-RVR (TATV) (Gao et al., 2017; Li et al., 2018; Zhong et al., 2018). Orthologues from diverse bacteria species have been discovered such as Mb3Cpf1, BsCpf1, and TsCpf1 (Table 1). The utility of Cas12a in genome editing is expending (Zetsche et al., 2015, 2017; Teng et al., 2019). A specific group of class 2 type V CRISPR enzyme named as Cms1 was identified from *Microgenomates* and *Smithella*. They are smaller than Cpf1, recognize AT-rich targeting sequence with PAM like TTN (SmCms1) and make cleavage without requirement of a trans-activating crRNA. Successful GE with Cms1 was confirmed in rice (Begemann et al., 2017). A distinct type V-B system from *Alicyclobacillus acidiphilus* (AaCas12b) (formerly known as C2c1) has also been functionally defined and adapted to editing mammalian genomes, in which the nuclease is able to active at temperature between 31 to 59°C (Teng et al., 2018). Similar to Cpf1, Cas12b recognizes a distal 5′-T-rich PAM, but it requires both crRNA and tracrRNA for target cleavage. Like in Cas9 system, a single guid RNA can be engineered for Cas12b (Cong et al., 2013). More and more Cas enzyme orthologous have been discovered with specific PAM recognition and cleavage outcomes such as CasX (known as Cas12c) with a 5′-TTCN PAM and an overhang of approximately 10nt at sticky-end, a deactivated CasX with a mutations introduced to the RuvC domain and CasY (also known as Cas12d) with a 5′-TA PAM recognition and dsDNA cleavage (Burstein et al., 2017; Liu J. J. et al., 2019). Cas13a (formerly C2c2) a class 2 type VI CRISPR system is characterized and modified to target RNA precisely (Abudayyeh et al., 2016, 2017). Unlike Cas9, Cas13a owns 2 enzymatically distinct ribonuclease activities required for RNA degrading process. One is to catalyze crRNA maturation, whereas the other RNase is responsible to make RNA-guided single-stranded RNA (ssRNA) cleavage using the catalytic sites in the two separate domains of higher eukaryote- and prokaryote-binding (HEPN). Cas13 variants have been identified, such as LshCas13a with a protospacer flanking sequence (PFS) of H (H denotes A, U or C) to recognize a 22–28nt target sequence, whereas LwaCas13a and PspCas13b without requiring specific PFS (Abudayyeh et al., 2017; Cox et al., 2017). The feasibility of Cas13a RNase activity for processing crRNA arrays make the system to target multiple RNAs simultaneously (Abudayyeh et al., 2016). Most of these Cas enzymes have been used in plant GE (Chen K. et al., 2019; Zhang Y. et al., 2019). For instance, Cas13a has been used to plant virus resistance (Aman et al., 2018; Zhang T. et al., 2019).

### An Invention of Cytidine or Adenine Base Editors and Development of New Base Editors

Base editing systems, consist of cytidine base editors (CBE) and adenine base editors (ABE), depend on CRISPR system and can make specific base changes without involving DNA DSBs and going through HDR pathway with a donor. The cytosine base-editor (CBE) system, composed of an catalytically inactive CRISPR/Cas9 domain including a guide RNA and a Cas9 nikase (nCas9) or dead Cas endonuclease (dCas9) fused with a cytidine deaminase inhibitor, makes conversion of a targeted cytosine into an uracil at targeting site in genomic DNA, which subsequently is replaced by a thymine during a DNA synthesis (Komor et al., 2016; Figure 1). CBE1 is the original cytosine base editor with which the desired cytosine at the targeting site in DNA is deaminized first and converted to uracil, leading to a U-G mismatch, which then can be repaired and substituted with a T-G in a newly synthesized strand through DNA repair pathway. Based on CBE1, an uracil glycosylase inhibitor (UGI) is fused to the dCas9 or nCas9 that inhibits uracil DNA glycosylase and prevent the transformation of uridine into an apurinic/apyrimidinic site, which makes CBE2. CBE3 is constructed with 2 fusion domains of a nickase Cas9 D10A, one with a rat cytosine deaminase rAPOBEC1 (apolipoprotein B mRNA editing enzyme, catalytic polypeptide-like) fused to its N terminus using a 16-amino acid XTEN linker and the other with a UGI fused to the C terminus using a 4-amino acid linker (Komor et al., 2016). The significant increase of the base conversion efficiency using BE3 is mainly due to substitution of the Cas9 in BE2 with a nickase dCas9 (nCas9) which nicks the untargeted strand in BE3. Based on BE3, BE4 (*S. pyogenes* Cas9-derived SpBE4 and *S. aureus* Cas9-derived SaBE4) is made by replacing the 16 aa linker with a 32-aa linker for rAPOBEC1 fused to Cas9D10A and using a 9-aa linker to fuse UGI one each to C and N terminal of Cas9 nickase, respectively, which enable repairing the non-edited strand using the edited strand as a template in cells and reducing undesired by-products through inhibiting base excision repair by using UGI (Komor et al., 2016, 2017). Efficient targeted C-to-T base editing with expanded PAM recognition in CRISPR/Cas9 system has also been achieved by fusing nCas9 to other orthologues from cytidine deaminase family members including APOBEC1, activation induced cytidine deaminase (AID), *Petromyzon marinus* cytosine deaminase 1 (PmCDA1) and APOBEC3A (antiviral cytidine deaminases of the human APOBEC3 (hA3)) (summarized in Chen K. et al., 2019). The ABE system, composed of *Escherichia coli* TadA (transfer RNA adenosine deaminase) and dCas9 or nCas9 (D10A), makes targeted adenine (A) change to Guanine (G) base editing in genomic DNA. The first-generation ABE, ABE1.2, is developed by fusing the TadA, evolved from *E. coli* TadA which catalyzes adenine deamination, to a nCas9 (Gaudelli et al., 2017). The later generation ABEs are made using various TadA mutations such as TadA^∗^, and fusion of the heterodimeric TadA (TadA-TadA^∗^) with nCas9 (D10A) made modified ABEs including ABE7.10, enabling A to G targeted base editing with increased efficiency and specificity in a wide range of targets (Mishra et al., 2020). RNA base editors (RBE) are developed by combining a catalytically inactive Cas13 (dCas13) with a naturally occurring adenosine deaminase acting on RNA (ADAR) for programmable adenosine to inosine substitution in mammalian cells (Cox et al., 2017). The later RBE version such as REPAIRv2 shows high specificity than previous one. RBE has been used for editing mammalian cells but not yet applied in plants. Base editing including ABEs and CBEs have been adapted in plant genome editing and successfully applied for point mutations in most major crops and model plant species (Summarized in Chen K. et al., 2019; Zhang Y. et al., 2019; Mishra et al., 2020).

### Newly Developed Prime Editing Method

Recently David Liu’s group at Broad Institute of Harvard developed a new editing method based on CRISPR system. The new prime editing (PE) system is composed of a Cas9 nikase conjugated with a reverse transcriptase (RTase) and a prime editing guide RNA (pegRNA). The pegRNA contains a classic sgRNA with a Cas targeting spacer region, a primer binding site (PBS) for reverse transcription (RT) initiation and a RT template with edits for targeting DNA changes. The pegRNA leads the prime editor to the target site in genomic DNA, Cas9 nickase generates a nick adjacent to the PAM, RTase-mediated primer extension from the 3’ end of the nick using the RT template with edits for targeting DNA changes. The reverse transcriptase element reads the RNA extension following the sequence designed for mutation in the template and newly synthesized strand is able to incorporate the corresponding DNA nucleotides with edits into the target sequence (Anzalone et al., 2019; Figure 1). Prime editing can achieve all 12 possible base changes or small indels or some combination of all of these (Yang et al., 2019). There are few restrictions on the edited sequence with this method. Prime editing is also able to introduce precise single base substitutions in target sequences and achieve changes with all types which is hard for current base editors to accomplish. PE induces less off-site targeting changes compared to other GE platforms. The versatile and precise editing outcomes have been confirmed in rice and wheat (Li et al., 2020; Lin et al., 2020; Tang et al., 2020; Xu et al., 2020).

Since the CRISPR system established in plant in Li et al. (2013), Nekrasov et al. (2013), Shan et al. (2013), much progress in basic plant science and crop improvement have been made, and various editing outcomes can be achieved in plant with adoption of the new CRISPR approaches including CRISPR/Cpf1 and other orthologues (Zhang Y. et al., 2019), nucleotide substitution tools for base editing (Mishra et al., 2020) and prime editing (Li et al., 2020; Lin et al., 2020; Tang et al., 2020; Xu et al., 2020). CRISPR system has rapidly superseded the earlier editing systems because the CRISPR system-based technologies are robust, low cost, simple to operate, easy to use and were widely adopted in plants (Chen K. et al., 2019; Zhang Y. et al., 2019). These new approaches will accelerate crop breeding with designed and accurate gene modifications directly in an elite cultivar background. The applications of GE in genetic research and variety improvement of crops have been intensively reviewed (Petolino, 2015; Weeks et al., 2016; Chen K. et al., 2019; Zhang Y. et al., 2019).

## General Procedure of Genome Editing in Soybean and Factors for Success

**FIGURE 2:**
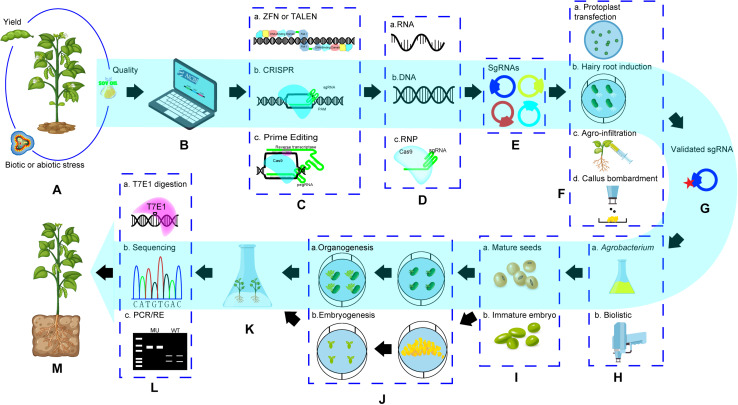
General procedure of soybean genome editing. **(A)**. Target trait selection; **(B)**. Search for bioinformation of genetic background of target traits and find out target genes for editing; **(C)**. Select a GE technology; **(D)**. Determine editing reagent form; **(E)**. Design and construct editing reagents; **(F).** Validate activity of putative GE reagents; **(G)**. a selected active editing reagent (construct) for GE; **(H)**. select a method for editing reagent delivery; **(I)**. Selecta explant; **(J)**. Go through a regeneration procedure based on regeneration pathway; **(K)**. Regenerated plants from explants transformed with editing reagents; **(L)**. Screening target gene edited events using molecular methods; **(M)**. A genome edited whole soybean plant. **(1)** Items in dotted box are the choice of technology platforms, method or explants. **(2)** Pathway in blue showed a GE procedure based on CRISPR system with DNA editing reagents and *Agrobacterium*-mediated delivery method.

In soybean, the first successful genome editing was done in hairy roots in which *GmDcl4a* and *GmDcl4b* genes were targeted using ZFNs (Sander et al., 2011). The first fertile GE soybean plants with mutation of *GmDcl4* gene (either *GmDcl4a* or *GmDcl4b*) was also created using ZFNs (Curtin et al., 2011). Haun et al. (2014) reported the first TALENs mediated GE events with 2 target sites simultaneously. The first successful CRISPR GE in soybean was reported in Jacobs et al. (2015). Initially most work with CRISPR/Cas9 focused on establishing GE system and evaluating its targeting efficiency in hairy roots (Cai et al., 2015; Jacobs et al., 2015; Michno et al., 2015; Sun et al., 2015) and the multiplex property with CRISPR to targeting pairs of genes simultaneously was also confirmed (Table 2). Meanwhile, the success of target gene knockout (Jacobs et al., 2015) as well as homology-directed recombination (HDR) in whole plants was achieved (Li et al., 2015). Since then, CRISPR has been used as a major method for soybean genome editing (Table 2). Cpf1 was used in soybean by Kim et al. (2017), who created mutations successfully in *FAD2* paralogues using CRISPR/Cpf1 RNP system and provided possibility to recover edited soybean events without involvement of DNA integration from reagents with plasmids, suggested a future direction for GE application in soybean. Like the trend of GE platforms used in other crops, ZFNs and TALENs had very limited use in soybean, but the CRISPR system is the most popular tool and it has been used extensively in soybean for functional genomic study and trait improvement (Table 2). A general procedure to recover GE events in soybean is illustrated in Figure 2. The key steps and factors affecting its success are:

**TABLE 2:** List of soybean genes edited for functional genetics study and trait improvement using genome editing technology.

Trait	Gene/Targeting location	Promoter of SgRNA	Promoter of Nucleases	GE plateform	Delivery method	Edited events	Editing outcomes	References
**Yield**								
Plant architecture	*GmLHY/*(GmLHY1a, GmLHY1b, GmLHY2a, GmLHY2b)	AtU3b/U3d AtU6-1/U6-29	CaMV35S	CRISPR/Cas9/	*A. tumefaciens*	Whole plant	Knockout (multiplex)	Cheng et al., 2019
	*GmSPL9/*(GmSPL9a, GmSPL9b, GmSPL9c, GmSPL9d)	AtU3b/U3d AtU6-1/U6-29	CaMV35S	CRISPR/Cas9	*A. tumefaciens*	Whole plant	Knockout (multiplex)	Bao et al., 2019
	*GmAP1/*(GmAP1a, GmAP1b, GmAP1c, GmAP1d)	AtU3b/U3d AtU6-1/U6-29	CaMV35S	CRISPR/Cas9	*A. tumefaciens*	Whole plant	Knockout (multiplex)	Chen et al., 2020b
Photoperiod	*GmFT2a*	AtU6	CaMV 2 × 35S	CRISPR/Cas9	*A. tumefaciens*	Whole plant	Knockout	Cai et al., 2018
	*GmFT2a and GmFT5a*	AtU6	CaMV 2 × 35S	CRISPR/Cas9	*A. tumefaciens*	Whole plant	Knockout (multiplex)	Cai et al., 2020b
	*GmFT2b*	AtU6	CaMV 2 × 35S	CRISPR/Cas9	*A. tumefaciens*	Whole plant	Knockout	Chen K. et al., 2019
	*GmE1 (Glyma.06G207800)*	AtU6	CaMV 2 × 35S	CRISPR/Cas9	*A. tumefaciens*	Whole plant	Knockout	Han et al., 2019
	*GmFT2aand GmFT4*	AtU6	CaMV 2 × 35S	BE base editor	*A. tumefaciens*	Whole plant	Base editing	Cai et al., 2020a
	*GmPRR37*	AtU6	CaMV 2 × 35S	CRISPR/Cas9	*A. tumefaciens*	Whole plant	Knock-out	Wang L. et al., 2020
	*GmAP1/*(GmAP1a, GmAP1b, GmAP1c, GmAP1d)	AtU3b/U3d AtU6-1/U6-29	CaMV35S	CRISPR/Cas9	*A. tumefaciens*	Whole plant	Knockout (multiplex)	Chen et al., 2020b
**Nutrition and quality**								
Storage protein	*Glyma.20g148400, Glyma.03g163500 Glyma.19g164900*	AtU6	ZmUbi	CRISPR/Cas9	*A. tumefaciens*	Whole plant	Knockout	Li C. et al., 2019
Seed oil	*GmFAD2-1A and GmFAD2-1B*		CaMV35S	TALENs	*A. rhizogenes*	Whole plant	Knock-out	Haun et al., 2014
	*GmFAD2–1A, GmFAD2–1B, GmFAD3A*		CaMV35S	TALENs	*A. tumefaciens*	Whole plant	Knock-out	Du et al., 2016
	*GmFAD2–1A, GmFAD2–1B*	AtU6	CaMV 2 × 35S	CRISPR/Cas9	*A. tumefaciens*	Whole plant	Knock-out (multiplex)	Do et al., 2019
	*GmFAD2–1A, GmFAD2–2A*	AtU6	CaMV 2 × 35S	CRISPR/Cas9	*A. tumefaciens*	Whole plant	Knock-out (multiplex)	Wu et al., 2020
	*GmFAD-2*	AtU6	CaMV35S	CRISPR/Cas9	*A. tumefaciens*	Whole plant	Knock-out	al Amin et al., 2019
Bean flavor-free soybean	*GmLox1, GmLox2, GmLox3*	GmU6	Gm4	CRISPR/Cas9	*A. tumefaciens*	Whole plant	Knock-out	Wang J. et al., 2020
**Abiotic stress tolerance**								
Herbicide resistance	*Als*	Gm (U6-9-1)	GmEF1A2	CRISPR/Cas9	Biolistic method	Whole plant	Knock-in (HDR)	Li et al., 2015
	*aad-1(2,4-D tolerance marker), dgt-28* (glyphosate tolerance marker) and *dsm-2 (glufosinate tolerance marker) at GmFAD2-1a locus*		Agrobacterium MANNOPINE SYNTHASE promoter	ZFNs	Biolistic method	Whole plant	Knock-in (NHEJ)	Bonawitz et al., 2019
**Nitrogen fixation**								
Root nodulation	*GmRIC1(Glyma.13G292300) andGmRIC2 (Glyma.06G284100), GmRDN1-1(Glyma.02G279600), GmRDN1-2(Glyma.14G035100) and GmRDN1-3 (Glyma.20G040500)*	GmU6	GmPm4, GmPm8	CRISPR/Cas 9	*A. tumefaciens*	Whole plan*t*	Knockout (multiplex)	Bao et al., 2019

**Gene function/Transgene for GE test**	**Gene/Targeting location**	**Promoter of SgRNA**	**Promoter of Nucleases**	**GE plateform**	**Delivery method**	**Edited event**	**Editing outcomes**	**References**

GE platform adoption in soybean	*GmDCL4a and GmDCL4b*		CoDA	ZFNs	*A. rhizogenes*	Hair root	Knockout	Sander et al., 2011
	*GFP Transgene, GmDCL1a, GmDCL1ab, GmDCL4a and GmDCL4b, GmRDR6a, GmRDR6b and GmHEN1a*		CoDA	ZFNs	*A. rhizogenes*	Hair root	Knockout	Curtin et al., 2011
	*Bar trans gene, GmFEI2 and GmSHR*	AtU6	ZmUbi	CRISPR/Cas 9	*A. tumefaciens*	Hair root	Knockout (Multiplex)	Cai et al., 2015
	*GmGS* (*Glyma18g04660 and Glyma.18g041100*), GmCHI20 (*Glyma20g38560 and Glyma.20g241500*)	AtU6-26	CaMV 2 × 35S	CRISPR/Cas9	*A. rhizogenes*	Hair root	Knockout	Michno et al., 2015
	*GFP* transgene,*01gDDM1, 11gDDM1, Glyma04g36150, Glyma06g18790, miR1509, and miR1514*	Mt U6.6	CaMV 2 × 35S	CRISPR/Cas9	*A. rhizogenes*	Hair root	Knockout	Jacobs et al., 2015
	*Glyma06g14180, Glyma08g02290 and Glyma12g37050)*	GmU6-10	CaMV35S	CRISPR/Cas9	*A. rhizogenes*	Hair root	Knockout	Sun et al., 2015
	*GmPDS11 and GmPDS18*		/	TALENs	*A. tumefaciens*	Hair root	Knockout	Du et al. (2016)
	*GmPDS11 and GmPDS18*	AtU6-26 GmU6-16g-1	ZmUbi	CRISPR/Cas9	*A. tumefaciens*	Whole plant	Knockout	Du et al. (2016)
	*FAD2-1A(Glyma10g42470) andFAD2-1B (Glyma20g24530)*	/	/	CRISPR/AsCpf1 or LpCpf1	Protoplast transfection	Protoplast	Knockout (RNP)	Kim et al., 2017
Egg cell promoter driving Cas9	*Promoter (GmEC1.1p, GmEC12p, AtEC1.2e1.1p, AtP5) and target gene GmAGO*	CaMV 2 × 35S	CaMV2 × 35S; AtEC1.2e1.1; GmEC1.1; GmEC12; AtP5	CRISPR/Cas9	*A. rhizogenes, A. tumefaciens*	Hair root and whole plant	Knockout	Zheng et al., 2020
Targeted deletions of DNA fragments	*GmFT2a* (Glyma16g26660) and *GmFT5a* (Glyma16g04830)	AtU6	CaMV 2 × 35S	CRISPR/Cas9	*A. tumefaciens*,	Whole plant	Knockout (4.5kb in GmFT2a)	Cai et al., 2018
Growth of soybean trichomes	*A. thaliana CPR5 ortholog* GmCPR5 (gene model Glyma.06g145800)	MtU6,	GmUbi-3P	CRISPR/Cas 9	Biolistic method	Whole plant	Knockout	Campbell et al., 2019
Seed weight and organ size	*GmPPD1 and GmPPD2*	GmU6-10	PcUbi	CRISPR/Cas 9	*A. tumefaciens*	Whole plant	Knockout	Kanazashi et al., 2018
miRNA pathway and Small RNA processing	*GmDCL4a and GmDCL4b, GFP*		Estrogen inducible promoter CoDA	ZFNs	*A. rhizogenes*	whole plant	Knockout	Curtin et al., 2011
	*GmDRB2(Gmdrb2a and Gmdrb2b), GmDCL3a, GmHEN1a* and *GmHEN1b*	At7sL AtU6	GmUbi AtUBQ10	CRISPR/Cas 9	*A. rhizogenes*	whole plant	Knockout	Curtin et al., 2018
	*GmDCL2b*		Arh rolD	TALENs	*A. rhizogenes*	whole plant	Knockout	Curtin et al., 2018
Sucrose export related embryo development	*GmSWEET15a and GmSWEET15b*	GmU6	CaMV 2 × 35S	CRISPR/Cas 9	*A. tumefaciens*	Whole plant	Knockout	Wang S. et al., 2019
circadian rhythmicity	*GmLCLa1, LCLa2, LCLb1, and LCLb2*	AtU3b/U3d AtU6-1/U6-29	CaMV35S	CRISPR/Cas 9	*A. tumefaciens*	Whole plant	Knockout (Multiplex)	Wang Y. et al., 2019
Soybean knockout library	70 sgRNAs to target 102 genes	GmU6	Gm4	CRISPR/Cas 9	*A. tumefaciens* (pooled)	Whole plant	Knockout (Multiplex)	Bao et al., 2019

### Selection of a Target Trait (Figures 2A,B)

The function and property of the genes controlling the target trait should be fully understood, which includes sequence data, transcription data, copy number in target materials and variations compared with reference genome. Soybean genome sequencing and gene discovery paves the way for GE. Prediction of more than 46,000 genes in the soybean genome has been done based on a soybean reference genome assembly using DNA sequences of Williams82 (Schmutz et al., 2010)^2^. Recently, hundreds of accessions of *G. max* and allied species have been sequenced for more reference genomes including the recent assembly high-quality reference genome of a wild soybean W05 and a popular Chinese cultivated soybean Zhonghuang 13 (ZH13) (The Genome Warehouse^3^) (Kim et al., 2010; Lam et al., 2010; Chung et al., 2014; Li B. et al., 2014; Li Y. H. et al., 2014; Zhou et al., 2015; Gao and Gao, 2017; Asaf et al., 2018; Shen et al., 2018; Xie et al., 2019). Moreover, hundreds of regulatory non-coding RNA loci, such as loci for microRNAs (miRNAs) and phased small interfering RNAs (phasiRNAs), have also been characterized using the soybean reference genome assemblies (Arikit et al., 2014). All of the sequence information can be evaluated using comparative genomics to identify potentially useful genes. Since soybean is a paleopolyploid and the two duplication events occurred 59 and 13 million years ago, respectively, more than 70% of these genes have been duplicated and exist as multiple copies. It is difficult to identify genes associated with important agronomical traits such as yield, protein, oil, as well as biotic and abiotic stress tolerances, which often makes soybean breeding programs complicated (Shoemaker et al., 2006; Yin et al., 2013; Zhu et al., 2014; Lakhssassi et al., 2017; Anguraj Vadivel et al., 2018; Chen K. et al., 2019). Therefore, trait selection for soybean genome editing depends on discovery of the genes which control important agronomic traits. The main challenge facing researchers for soybean improvement has been the limitation of understanding the functions of genes and their contributions to target phenotypes of agronomic importance. Based on the current knowledge, GE in soybean has focused on traits with clear genetic background such as *GmFAD2* for oleic oil content.

### Selection of One GE Technology and Preparation of Editing Reagents Related to the Choice of GE Platform (Figures 2C-G)

Except for ZFNs and TALENs which are not popular due to their technique complexity, high cost and inflexibility in use, CRISPR/Cas9 system is becoming the most efficient GE technology for soybean. Related system such as CRISPR/Cpf1 and others could be an alternative due to their simplicity and easy operation property. Two editing reagents, including sgRNA and one of related nuclease proteins such as Cas9, Cpf1, various Cas9 orthologous and Cas proteins, are prerequisite for GE. The Cas gene and the target sgRNA can be constructed in one plasmid or each of them in a separate plasmid as shown in previous reports (Jiang et al., 2013; Li et al., 2013; Brooks et al., 2014). In most cases in CRISPR system for soybean, the *Streptococcus pyogenes* Cas9 gene (Cai et al., 2015; Cheng et al., 2019), human codon-optimized Cas9 gene (Campbell et al., 2019; Do et al., 2019) or a soybean codon optimized Cas9 gene (Michno et al., 2015; Sun et al., 2015) was driven by commonly used promoters in soybean transformation, such as ubiquitin (soybean ubiquitin-3, Campbell et al., 2019), Arabidopsis egg-cell specific promoter (Zheng et al., 2020) and cauliflower mosaic virus 35S (CaMV 35S) promoters (Cai et al., 2015; Jacobs et al., 2015; Michno et al., 2015; Bao et al., 2019; Chen F. et al., 2019; Cheng et al., 2019; Do et al., 2019; Han et al., 2019). Some endogenous promoters, such as proGmSCREAM M4 (pM4), proGmSCREAM M8 (pM8) (Bai et al., 2020) and soybean ELONGATION FACTOR1 ALPHA2 (EF1A2) gene constitutive promoter (Li et al., 2015), were used as well. Nuclear Localization Signal (NLS) sequences and Flag peptide sequences were normally inserted in each end of the Cas9 gene, which facilitate Cas9 protein entering the nucleus and protein identification. Because plant specific RNA polymerase III promoters such as AtU6 (Arabidopsis), TaU6 (wheat), and OsU6 or OsU3 (rice) are frequently used to drive gRNA in plant systems, dicotyledons U6 promoter such as Arabidopsis AtU6 (Cai et al., 2015; Michno et al., 2015; Do et al., 2019; Han et al., 2019), as well as *Medicago truncatula* MtU6 (Jacobs et al., 2015; Campbell et al., 2019), were frequently used to drive sgRNA for soybean. Soybean GmU6 promoters have been discovered and evaluated for their efficiency and used for GE (Li et al., 2015; Du et al., 2016; Di et al., 2019). For example, Di et al. (2019) tested 11 GmU6 promoters in soybean and Arabidopsis and found that GmU6-4, 7, 8, 10 and 11 had high performance. Compared to the AtU6-26 promoter, soybean GmU6-16-1 promoter was more efficient in simultaneous editing of multiple homoeoalleles (Du et al., 2016). sgRNA module vectors for soybean are usually based on common transformation vectors either for *Agrobacterium*-mediated delivery or biolistic delivery methods. For multiplex, more than two sgRNA expression cassettes could be assembled in each of these vectors (Du et al., 2016; Do et al., 2019).

Usually, a number of engineered editing reagents for ZFNs, TELENs, or CRISPR/Cas systems, does not show editing activity and cannot create any mutation event *in vivo* without knowing any cause (Li R. et al., 2019). Therefore, identification of mutations *in vivo* by validating sgRNA expression and nuclease activity for specific editing reagents *via* transient assay could save time and resources, and increase success rate before transformation for creating genome edited plants (Do et al., 2019; Bai et al., 2020). This step is especially important for those complicated GE platforms such as ZFNs and TALENs. Transient expression systems such as the hairy root induction system *via Agrobacterium rhizogenes* K599, callus tissue expression *via* biolistic delivery, agro-infiltration using leaves and protoplast transfection system, have been employed for the purpose (Figure 2). The assay for editing activity in hairy roots is a popular transient system for soybean (Curtin et al., 2011; Haun et al., 2014; Jacobs et al., 2015; Du et al., 2016; Cheng et al., 2019; Do et al., 2019; Bai et al., 2020), *A. rhizogenes* K599 containing editing reagents were delivered into seedlings of target genotypes and hairy roots can be recovered within 15 days. Editing activity of the reagents can be evaluated in those hairy roots. For protoplast transfection, polyethylene glycol (PEG) is used to deliver editing reagents into soybean protoplasts and then the targeted mutation events can be detected in genomic DNA extracted from the transfected protoplast after 48 h of incubation in darkness at room temperature (Sun et al., 2015; Demorest et al., 2016). Callus tissue is one of best target tissue for biolistic delivery and the transformed callus cells can be harvested within 5 weeks for evaluating GE including HDR events (Li et al., 2015; Bonawitz et al., 2019

### System for Recovering Target Gene Edited Whole Plants (Figures 2H-K)

The success of GE in soybean is highly dependent on availability of an efficient regeneration and transformation system. Like in other crops, biolistic and *Agrobacterium*-mediated soybean transformation methods have mainly been used to recover GE events (Table 2). *Agrobacterium*-mediated soybean transformation combining organogenesis-based regeneration is developed for soybean transgenesis and the transformation efficiency (TE) in several protocols has been improved (Yamada et al., 2012; Li et al., 2017; Hada et al., 2018; Yang et al., 2018), but it is still low (∼10%) compared to the high efficiency in rice (∼40%) (Mohammed et al., 2019). Cotyledons of soybean mature seeds were usually utilized as explants for regeneration of transformed cells. However, genotype dependency is still the major bottleneck among these protocols. For SDN1 mutations in soybean, *Agrobacterium*-mediated transformation system is currently an efficient and popular method (Table 2) due to convenient mature seed used as explants. GE with multiplex have been achieved in soybean to recover simultaneously target multiple genes or genomic sites in a single transformation event through either making one construct with the expression of multiple sgRNAs using a single Cas9 (Bao et al., 2019; Do et al., 2019) or infecting target tissue with pooled *Agrobacterium* strains each containing one sgRNA (Kanazashi et al., 2018; Cheng et al., 2019; Do et al., 2019; Wang S. et al., 2019; Wang Y. et al., 2019; Wang J. et al., 2020; Bai et al., 2020). Biolistic delivery plus embryogenesis-based regeneration system is an alternative method to recover GE evens with various outcomes. In soybean, all SDN2 and SDN3 currently reported were created using this method (Table 2). Availability to deliver multiple editing reagents such as pooled sgRNA constructs and RNP (RNA and protein complex), and large quantity of donor DNA fragments may have facilitated the achievement of SDN2 and SDN3. Biolistic transformation in soybean is highly genotype dependent (Homrich et al., 2012) since the regeneration is based on embryogenic suspension initiated from immature embryo of the target genotype such as Jack (Campbell et al., 2019) and 3B86 (Li et al., 2015). Although embryonic axes can be used for biolistic transformation, the efficiency is very low (Rech et al., 2008). *A. rhizogenes*-mediated transformation for recovery of GE whole plant was occasionally used (Curtin et al., 2011; Haun et al., 2014) and specific regeneration system need to be established for this method.

### Screening of Mutation Events Created by GE (Figures 2L-M)

Generally, the genomic DNA was extracted from transgenic soybean plants or plants regenerated from explants in which GE reagents was delivered. PCR primers were designed to amplify an amplicon containing the target sequence. PCR/RE assay (PCR products containing target sequence region with a restriction enzyme (RE) cut site cannot be digested with the RE if target editing is success), T7EI (T7 endonuclease I) assay and sequencing are commonly used to identify GE events (Shan et al., 2014). PCR/RE assay detection method was used frequently in soybean (Michno et al., 2015; Sun et al., 2015; Kanazashi et al., 2018; Di et al., 2019). T7EI assay was occasionally used (Cai et al., 2015; Du et al., 2016) and PCR/Sequencing assay is now the most popular method used to detect mutation in soybean (Haun et al., 2014; Campbell et al., 2019; Cheng et al., 2019; Do et al., 2019; Han et al., 2019; Li R. et al., 2019; Wang S. et al., 2019; Wang J. et al., 2020). PCR/RE and T7EI are cost efficient method when large number putative mutations need to be screened, but limitation of restriction enzyme cut sites can restrict design of sgRNA. Therefore, the sequencing-based method is a popular way to detect any target site in genome.

CRISPR system is efficient to induce mutation both in soybean hairy roots and regenerated plants. The editing frequency could reach up to 61.41% in hairy roots (Bai et al., 2020) and 72.20% in T0 generation (Cai et al., 2018). However, Bao et al. (2019) reported low efficiency in T0 plants. It may be to do with sequence composition at target site, sgRNA design, Cas9 codon optimization, and construction of GE reagents. Various type mutations including biallelic, homozygous (individuals homozygous/biallelic for all copies of the allele mutated will display a mutant phenotype), heterozygous (individuals heterozygous with at least one copy of the wild type allele will not display a mutant phenotype) and chimeric mutation can be obtained in soybean. GE events in most soybean T0 plants could be transmitted to next generation, but some may be lost in T2 plants (Do et al., 2019). To date, a couple of protocols for soybean genome editing based on CRISPR have been published, including one for recovery of GE whole plants (Liu J. et al., 2019) and one for GE hairy roots (Alok et al., 2018).

## GE Applications for Functional Genomics and Trait Improvement

### Achievement of Various Editing Outcomes

Most editing outcomes, including large fragment deletion, multiplexing, base editing, HDR editing, HDR insertion and knockout any given endogenous gene or genomic site, have been achieved in soybean (Table 2). Cai et al. (2018) designed a dual CRISPR sgRNAs and successfully deleted targeted DNA fragments in both soybean *GmFT2a* and *GmFT5a* gene. Fragments varying between 599 to 1618 bp in *GmFT2a* was deleted with a 15.6% frequency and 1069 to 1161 bp in *GmFT5a* were achieved with 15.8%. Furthermore, a target fragment larger than 4.5kb in *GmFT2a* were also deleted with a 12.1% frequency. Multiplex with various range of target sites have been made (Cai et al., 2015; Jacobs et al., 2015; Kanazashi et al., 2018; Bai et al., 2020). For example, Bao et al. (2019) assembled four sgRNAs driven by the AtU3 or AtU6 promoter in one binary CRISPR/Cas9 plasmid and achieved simultaneous targeting multiple sites in four genes in SPL9 (Squamosa Promoter Binding Protein-Like (SPL)) transcription factor family in soybean, and plants carrying various combinations of mutations including homozygous quadruple mutants in T4 generation were recovered using *Agrobacterium*-mediated transformation. Base editing at target sequences in the first exon of soybean flower control gene *GmFT2a* and fourth exon of *GmFT4* was successfully achieved using BE base editor combined the Cas9n (D10A) nickase, rat cytosine deaminase (APOBEC1), and uracil glycosylase inhibitor (UGI) (Cai et al., 2020a). Both C-T and C-G base substitutions was obtained but only the side effect C-G substitution in *GmFT2a* gene made the proline of its amino acid changing to alanine in the mutant, resulting in altered flowering phenotype. Homologous directed recombination has been achieved for both precise gene editing and site-specific knock-in using biolistic delivery (Li et al., 2015). A directed P178S mutation of acetolactate synthase1 (ALS) gene in soybean was made through HDR using a donor DNA template with a 1,084-bp *ALS1* sequence fragment containing five nucleotides AG-T-C-T changes along with a construct containing ALS1-CR1 gRNA and Cas9 through co-transformation of soybean with chlorsulfuron selection. Meanwhile, precise homology-directed gene insertion by Cas9-gRNA was also achieved by co-transform soybean with a donor DNA construct carrying a hygromycin phosphotransferase (*hpt*) gene driven by a soybean S-adenosyl methionine synthetase (SAMS) gene promoter to confer hygromycin resistance and a Cas9-gRNA targeting a soybean genomic site DD43 on chromosome 4. The homologous HDR was transmitted into next generation. Bonawitz et al. (2019) reported integration of ballistically delivered DNA to a targeting site in *GmFAD2-1a* (the Fatty Acid Desaturase 2-1a) gene in soybean and demonstrated targeted integration of multiple transgenes into a single locus in soybean via either HDR or NHEJ using a ZFN. A hygromycin resistant gene *hpt* and its regulatory elements were inserted into the target site through HDR and a NHEJ-mediated accurate insertion was achieved with a 16.2kb donor containing four transgenes, *hpt, dgt-28* (glyphosate tolerance marker), *aad-1* (2,4-D tolerance marker) and *dsm-2* (glufosinate tolerance marker). These integrations in T0 plant was successfully transmitted to T1 generation. Success of DNA-free GE with Cpf1 RNP was demonstrated in soybean protoplast (Kim et al., 2017).

### Editing Efficiency Improvement by Using Appropriate Promoters

Promoter for sgRNA in CRISPR system is one of factors affecting GE efficiency. One major progress is the discovery of soybean U6 promoters for driving sgRNA. Although U6 promoters have highly efficient transcription, it is difficult to use the same U6 promoter among various distantly related species because endogenous sequences are less susceptible to silencing associated DNA methylation than transgene sequences in plants (Wang et al., 2008). Various transcription activities were discovered when the same U6 promoter was used in divergent species (Shan et al., 2013). This effect was also confirmed in soybean (Sun et al., 2015), in which two types of vectors using either the GmU6-10 or AtU6-26 promoter were constructed to target several soybean genes. Significant different mutation efficiencies, 3.2-9.7% with AtU6 vector and 14.7-20.2% with GmU6-10 vector, were observed. Even the different U6 promoters from the same species showed various activities (Domitrovich and Kunkel, 2003). Soybean U6 promoters (GmU6-8 and GmU6-10) with high editing efficiency have been selected from 11 candidate promoters in hairy roots (Di et al., 2019). Du et al. (2016) compared targeting efficiency using both TALENs and CRISPR to knock out both *GmPDS11* and *GmPDS18* in hairy roots. In CRISPR/Cas9, when AtU6-26 promoter was used, the single targeting efficiency was similar to that achieved by TALENs. The efficiency was doubled by using GmU6-16g-1. Meanwhile, using the AtU6-26 and GmU6-16g-1 promoter in CRISPR/Cas9 achieved targeting efficiency 2 times and 8 times higher, respectively, than that by TALENs, indicating high efficiency of GE can be achieved by CRISPR system if an appropriate promoter is used to drive sgRNA. It is the fact that use of the DD45 (egg cell and early embryo), Yao (shoot apical and root meristem-active), tomato Lat52 (pollen) and EC (egg cells, embryo) promoters for driving Cas9 can reduce the frequency of somatic mutations and increases the rate of heritable edits in the T2 generation (Wang et al., 2015; Yan et al., 2015; Mao et al., 2016). Zheng et al. (2020) used both Arabidopsis and soybean egg-cell specific promoters to create knockout mutation of GmAGO7a gene in soybean. Successful mutations with T2 generation was achieved using AtEC1.2e1.1p promoters, but no mutants were recovered with GE using soybean egg cell promoters.

### Functional Genomics Study

Functions of many genes in soybean have been evaluated using GE (Table 2). For example, mutations for genes involved in small RNA processing were created using both CRISPR and TALENs for evaluating the role of small RNA processing in stress tolerance in soybean. CRISPR/Cas9 was employed to generate a biallelic double mutant of the two paralogous Double-stranded RNA-binding2 (*GmDRB2a* and *GmDRB2b*) genes, a heterozygous mutant for Dicer-like3 gene (*GmDCL3*a) and the homoeologous mutations of soybean Hen1 locus (*GmHen1a*; *GmHen1b*), and TALENs was used to induce mutant for dicer-like gene *GmDCL2b*. Some of the mutants in T0 plants can transmitted into T1 generation (Curtin et al., 2018). Li C. et al. (2019) reported successful targeting 3 different genes encoding two major storage protein families, conglycinins (7S) and glycinins (11S) accounting for about 70% of total soybean seed protein, and detected DNA mutations at a ratio ranging from 3.8 to 43.7% in the three storage protein genes in soybean hairy roots. Again Li R. et al. (2019) used pooled CRISPR/Cas9 technique to create single and double mutants of 2 plastidial phosphoglycerate kinase *PGKp1* and *PGKp2* gene. Normal performance of the single mutants and lethal phenotype of the double mutant confirmed that *PGK*s play redundant role in carbon fixation and metabolism. Paralogous sugar transport gene *GmSWEET15a* and *GmSWEET15b* from the SWEET (Sugars Will Eventually be Exported Transporter) family in soybean were targeted using CRISPR/Cas9 (Wang S. et al., 2019) and the knockout mutations showed abnormal growth of embryo and persistent endosperm, leading seed abortion. Multiplex mutagenesis populations for gene function evaluation was also created in soybean using a pooled CRISPR-Cas9 platform (Bai et al., 2020). Soybean was transformed with pooled *Agrobacterium* strains each containing one of 70 vectors with gRNA to target 102 candidate genes (4 to 5 stains each batch) and all targeted mutations have been achieved.

### Modification of Agronomy Traits

Genome editing technology has been applied to edit various genes controlling soybean agronomic traits (Table 2). Soybean oil nutrition was improved by knocking out fatty acid desaturase gene (*GmFAD2-1A and B*) using TALEN. The fatty acid composition was significantly changed in Bert seeds of *fad2-1a1b* homozygous double mutation plants, oleic acid was increased 4 times and reached to 78% and linoleic acid was reduced to less than 4% from original 50% (Haun et al., 2014). The third fatty acid desaturase 3A (*FAD3A*) gene was mutated in the double *fad2-1a1b* mutant created by Haun et al. (2014) using TALENs, and linolenic acid and linoleic acid in seed oil of these plants with triple *fad2-1a1b3a* homologous mutations were further reduced nearly by a half (2.5 and 2.7%, respectively) and oleic acid was increased significantly (82.2%) compared to those in the double *fad2-1a1b* mutants (Demorest et al., 2016). Similar work done in variety Maverick using CRISPR, which resulted in dramatic increase in oleic acid content to over 80% and decrease in linoleic acid to 1.3-1.7% (Do et al., 2019). Double knockout of *GmFAD2-1A* (*Glyma.10G278000*) and *GmFAD2-2B* (*Glyma.19G147300*) in variety JN38 has resulted in increase of oleic acid content in seeds from 19.15% to 72.02%; decrease of linoleic acid from 56.58% to 17.27% in the T3 generation (Chen et al., 2011). Moreover, the percentage of protein in the seeds was increased from 37.52% to 40.58% (Wu et al., 2020). Seed lipoxygenase-free soybean was created by mutating three lipoxygenases genes (LOXs, including *LOX1*, *LOX2*, and *LOX3*) using CRISPR/Cas9 since beany flavor restricts human consumption of soybean (Wang J. et al., 2020). Soybean is a short-day (SD) plant and it tends to flower when the day length reduces to a certain extent. Therefore, photoperiod regulates soybean to initiate flowering and to adapt in different environment conditions. FLOWER LOCUS T (FT) encodes florigen which induces floral initiation at the shoot apex (Kardailsky et al., 1999). FT also integrates signals in flowering pathways to control flower time (Corbesier and Coupland, 2006; Turck et al., 2008). Soybean FT homologous genes including *GmFT2a* and *GmFT5a* have been recognized, and their basic functions especially the photoperiod responsive effect have been evaluated (Kong et al., 2010). These roles of the FTs in soybean need to be further confirmed by using reverse genetics. Cai et al. (2018) evaluated the function of *GmFT2a* by knockout this gene using CRISPR/Cas9 and found that the homozygous *GmFT2a* mutants delayed flowering in any photoperiod condition. Knockout *GmFT2b* also delays flowering time under long day (LD) conditions (Chen et al., 2020a). Moreover, both *GmFT2a* and *GmFT5a* were found to control flowering time collectively when single mutant plants ft2a and ft5a, and double mutants ft2aft5a were assessed together with transgenic plants overexpressing *GmFT2a* or *GmFT5a* in photoperiod conditions including SD and LD. *GmFT2a* plays more important role for flowering than that of *GmFT5a* under SD conditions, and vice versa for *GmFT5a* and *GmFT2a* under LD conditions (Cai et al., 2020a). Unlike *GmFT2a, GmFT5a*, make soybean for high latitude adaption. When grown under SD condition, the ft2aft5a double mutants delayed flowering by 31.3 days, leading significant increases in numbers of pods and seeds per plant (Cai et al., 2020b). The circadian clock related gene, LONG ELONGATED HYPOCOTYL (LHY) and CIRCADIAN CLOCK ASSOCIATED 1 (CCA1), regulates flowering under different daylength conditions (Wang and Tobin, 1998). LHY-CCA1-LIKE orthologs in soybean, *GmLCLa1, GmLCLa2, GmLCLb1, and GmLCLb2*, were identified and CRISPR/Cas9 was used to knock out all the 4 orthologs simultaneously to investigate their circadian rhythm related function in soybean (Wang Y. et al., 2019). The quadruple mutant *GmLCLa1a2b1b2* delays flowering and showed very short-period circadian rhythms. Early flowing mutations under natural long-day (NLD) conditions was also created by knockout *E1*gene (Han et al., 2019) and *GmPRR37* encoding a pseudo-response regulator protein which is related to photoperiod sensitivity using CRISPR system (Wang L. et al., 2020). These flowering time related mutations can be consistently inherited in next generation (Cai et al., 2018; Han et al., 2019; Cai et al., 2020a; Wang J. et al., 2020) and will be a useful resource for developing elite soybean varieties in the future. Plant architecture can be modified for improving yield. Cheng et al. (2019) mutated 4 Late Elongated Hypocotyl (LHY) genes again in soybean and obtained a homozygous quadruple mutant of *GmLHY* which is similar to *GmLCLa1a2b1b2* described above and showed reduced plant height and shortened internodes. As a class A gene in the ABCE model in plant, *APETALA1* (*AP1*) involves in floral organ development. All 4 soybean AP1 homologous genes have been targeted using CRSPR/Cas9 and the homologous quadruple gene knockout events delayed flowering time under SD and showed increase in plant height with increased node number and internode length, indicating potential yield increase for the mutation events (Chen et al., 2020b). Bao et al. (2019) mutated four gene encoding SPL transcription factors of the SPL9 family in soybean. Mutant plants carrying various combinations of mutations including a quadruple homologous mutant *Gmspl9ab-1cd* generally displayed significant plant architecture changes and showed various increases in total node number per plants at different levels depending on node number on the main stem and branch number. This result indicates each of the 4 genes play important role to regulate plant architecture. Bai et al. (2020) created targeted mutations in paralogous gene *GmRIC1* and *GmRIC2* that encode two nodule-enhanced Cavata3/Embryo Surrounding Region-Related (CLE) peptides using CRISPR. Two different types of double homozygous *gmric1/gmric2* mutant plants demonstrated significant nodule number increase. Meanwhile, mutants for soybean Root Determined Nodulation1 (*GmRDN1*) were created as well. Down-regulation of all three target genes in the triple mutant *gmrdn1-1/1-2/1-3* plants confirmed *GmRDN1* negative regulation of nodule numbers in the roots. CRISPR has also been used to create herbicide resistant soybean. An HDR directed P178S mutation of acetolactate synthase1 gene in soybean was created using CRISPR system, which is resistant to chlorsulfuron (Li et al., 2015).

CALYXT has performed field trials with GE soybean in Argentina since 2015 and launched its first commercial soybean variety edited by GE in 2018. This is the first GE soybean product in the world^4^.

## Challenges and Prospective for GE and Related Product Development in Soybean

Recent popular transgenic technology used in the last 4 decades has introduced foreign genes into crops including soybean for desired traits, and it has indeed made an alternative way to expand genetic resource. However, the random integration of transgenes in genome has raised public concerns and strict government regulation, which have dramatically increased cost and time for developing a new variety. GE technology provides a very efficient tool for crop breeders to introduce a desired trait into an elite background with precise and predictable manner rather than going through multiple back crossing to transfer a nature mutation in a typical conventional breeding process. The mutations created by GE is indistinguishable from these introduced by traditional mutagenesis breeding. This can also avoid the issues related to transgenic technology. Although various GE technology platforms have been extensively used in soybean and many editing outcomes can be created as summarized above, there are some difficulties. Similar to other crops (Scheben and Edwards, 2018), the biggest bottleneck for GE application in soybean is the deficit of GE candidate target genes due to insufficient fundamental study in soybean as stated above. The other bottlenecks include technical issues such as lack of guarantee to precise mutate any target site, the limitation of ways to deliver the genome-editing reagents into soybean cells, the low efficiency to select desired events and regenerate intact plants with targeted mutation, and off-site targeting. Many attempts have been made to minimize the limitations and improve efficiency to recover GE events through using newly developed GE technologies and soybean regeneration system. There are also some additional concerns for GE product development such as transgenic GE events, restriction of intellectual property and government regulation for GE. These issues need to be resolved before GE can play an important role in soybean improvement.

### GE at Any Target Site in a Target Gene Sequence

This is a common GE issue for all plant crops. Most genome editing research using current available GE technologies still focus on gene knockout or generating a null mutation (SDN1). In most cases, multiplex editing is desired to overcome gene duplications in soybean due to its paleopolyploid nature, whereas single gene editing is used to resolve functional redundancy and unique role from each of the gene paralogs. Loss of gene functions can be easily identified from phenotypes or by molecular tools such as PCR. Due to lack of understanding of HDR mechanism and mature methods, only a couple of studies reported achieving SDN2 and SDN3 through CRISPR and ZFNs (Li et al., 2015; Bonawitz et al., 2019). New technology such as base editing has potential to achieve the same outcomes as SDN2. However, it has not been fully adapted in soybean despite a success base editing case with a BE base editor in soybean reported recently (Cai et al., 2020a). The less success of base editing in soybean highlight the need to develop this technology in soybean. PAM site dependence and editing window may be the key factors. The possible solution is to use new types of Cas proteins, engineering Cas variants with altered PAM and modify the linker between deaminase and nCAS9 (Chen K. et al., 2019; Mishra et al., 2020). Recently, another powerful GE technology-primer editing (PE) has been developed (Anzalone et al., 2019). Theoretically, it has possibility to make GE at any target site in a target gene sequence. To date, PE is used successfully in rice and wheat (Li et al., 2020; Lin et al., 2020; Tang et al., 2020; Xu et al., 2020). If the system can be fully operated in soybean, GE with all types of editing outcomes can be readily achieved.

### System to Recovery of GE Whole Plant From Any Type Explants of Any Genotype

Among editing reagent delivery methods, *Agrobacterium*-mediated method and biolistic method are commonly used for GE in soybean with all available GE platforms (Table 2). These systems depend on tissue culture procedure with either organogenesis, i.e., multiple shoots regenerated from embryonic cotyledons of mature seeds, or embryogenesis, i.e., shoots regenerated from embryogenic callus derived from immature cotyledons (Yamada et al., 2012). The genotype dependent has been a very well know issue for *Agrobacterium*-mediated transformation since the method was implemented in soybean. It is also a big issue for using the biolistic method in soybean and the specific explant requirement for embryogenic tissue restricted application of GE in any genotype of soybean. Target genotype dependent, explant specificity and GE outcome dependent are the main limitations existed in current soybean transformation system for GE application. There are several ways to overcome the genotype dependent issue. Embryogenic booster genes such as *BBM* (*BABY BOOM*) and *WUS* (*WUSCHEL*) has been used in maize and other monocot plants to promote plant regeneration from various tissues (Lowe et al., 2016; Mookkan et al., 2017) and plant regeneration booster *GRFs* (*GROWTH-REGULATING FACTORs*)-*GIF1* (*GRF-INTERACTING FACTOR 1*) complex has been used in both monocot and dicot (Debernardi et al., 2020). This can potentially broad explant types and to break through the genotype limitation if the similar booster genes can be found to improve regeneration in soybean. The other way is to develop *in planta* transformation method, which does not depend on genotype, such as *A. thaliana* floral dip transformation. In-planta transformation methods has been developed in soybean (Mangena, 2019) but they are not ready for GE due to its current low efficiency. Improvement of *Agrobacterium*-mediated delivery method and transformation efficiency for recovery of various editing outcomes, development of other delivery and regeneration system such as protoplast transfection and regeneration system or *in planta* transformation system will expand the GE application in soybean.

### Off-Site Targeting

Off-site targeting is caused by introduction of unintended mutations at off-target sites during genome editing process (Hahn and Nekrasov, 2019). In plants, this issue is not considered as the same important as in mammals since abnormal off-site target mutations can normally be identified and discarded through offspring segregation using backcrossing. Nevertheless, remove of off-site targeting can be time consuming in plant breeding. For CRISPR system, different sgRNAs structure (Mali et al., 2013) and specificity of Cas9 such as high-fidelity SpCas9 variants (Zhang et al., 2017) can affect the cleavage on target and off targets sites. In soybean, off-site targeting was not detected in mutants created using ZFNs and TALENs, but it was evaluated and screened in edited events created using CRISPR system (Sun et al., 2015). For example, two possible off-target sites was detected in the genome of the soybean cultivar Williams82 using the web tool CRISPR-P^5^ when targeted mutation for FAD2 genes was designed using CRISPR (Do et al., 2019). The off-site targeting in plant can be avoided through evaluating and predicted using various web-based tools such as Cas-OFFinder (Bae et al., 2014), CROP-IT (Singh et al., 2015), CRISPOR (Haeussler et al., 2016), and other soft tools (Hahn and Nekrasov, 2019). The effect can be reduced by improving specificity of CRISPR system using high-fidelity SpCas9 variants (Zhang et al., 2017; Zhong et al., 2019), nCas9 (nickase) with two sgRNAs (Shen et al., 2014; Zhang et al., 2015), delivering purified Cas9 ribonucleoproteins (RNPs) into cells (Kim et al., 2017; Andersson et al., 2018), and modified sgRNA (Young et al., 2019). For soybean, there has been a system established for the scientific society to shared genome-wide databases and to identify off site targets (Zou et al., 2020). In this system, specificity score and off-target number for each CRISPR/Cas9 targeting site can be calculated and evaluated, which would help to minimize the off-site targeting for GE in soybean during its applications in the future.

### Transgene in GE Events

If GE product is not covered by genetically modified organism (GMO) regulation, the cost of field test and data collections would be massively reduced. It could also dramatically save time in release GE product and will reduce the public concerns on consuming GMO crops. Therefore, transgene-free or DNA-free GE plants are a pre-request for product development. Generally, genome edited plants are transgenic plants since GE events are normally recovered using transformation system and the form of editing reagents is DNA. Using next generation sequencing (NGS) analysis, Michno et al. (2020) found that three different CRISPR/Cas9 transgenes and their respective induced mutations in segregating soybean families have both expected and unexpected patterns of inheritance in different progeny lines at T0 and T1 generation. However, it is possible to obtain GE events without transgene integration in next generations through segregation. Transgene-free events obtained in T2 or T3 generation through transgene segregation is the major way to have transgene-free GE plants in soybean (Haun et al., 2014; Wang S. et al., 2019). Some homozygous mutant soybean plants without transgene can be easily identified in the T1 mutant population such as *GmFAD2* GE soybean (Do et al., 2019). Like GE technologies used in other crops, RNA or RNA and protein complex (RNP) editing reagents can be used to obtain DNA-free GE soybean. This can avoid transgenic and off-site targeting issue since biolistic delivery method established in soybean can be used for this purpose (Woo et al., 2015; Svitashev et al., 2016; Liang et al., 2017; Andersson et al., 2018). Development of new transformation methods such as protoplast transfection or *in planta* transformation including various GE methods bypassing tissue culture (Ji et al., 2020) will guarantee achieving RNP-mediated DNA-free GE soybean product.

### Government Regulation for GE Product and Intellectual Property Preparation

It is one of major concerns for soybean breeders if GE is under same regulation framework as that of GMO. It normally based on socio-economic considerations rather than scientific evidence, which delays the adoption of GM crops leading to a negative impact on global agricultural innovation (Biden et al., 2018). In many countries, a variety developed through precise targeting mutation such as SDN1 created using technologies like ZFNs, TALENs, and CRISPR does not need to go through the regulation process used for GMO (Friedrichs et al., 2019; Metje-Sprink et al., 2020; Schmidt et al., 2020). GE plants and the products can be cultivated and sold free from regulatory monitoring in the United States, Brazil, Argentina, Chile, Australia, and Japan. In most countries, the regulation of GE plants is based on assessment of the product except for that in the EU, Brazil, Indian and New Zealand, which is dependent on the biotechnological processes to produce the organism. Several countries made decision to follow a product-based approach and some countries like Australia and China tend to follow in near future (Friedrichs et al., 2019; Metje-Sprink et al., 2020; Schmidt et al., 2020). Soybean breeders should collect such information and develop new varieties aiming mainly at countries which already have low or no regulation on GE plants or countries that will remove GE from GMO regulations. Another concern is GE intellectual property for commercial soybean product. Selecting one of the highly efficient, easy to operate and low-cost technology will accelerate GE product development for soybean. In contrast to other genome editing techniques such as ZFNs and TALENs, which had clear intellectual property ownership, CRISPR does not have clear ownership yet. Several institutes and companies have claimed rights to this system and this issue currently remains unresolved (Brinegar et al., 2017). Since its development, the number of patents related to CRISPR products has increased at an unprecedented rate compared to other editing technologies, such as CPF1, CMS and a recently developed Prime editing technology which have been successfully used in plant (Li et al., 2020; Lin et al., 2020; Tang et al., 2020; Xu et al., 2020).

## Conclusion Remarks

Precise and predictable modifications of desired targeting gene sequences in an elite background without change other traits by genome editing can accelerate plant breeding. In crops with duplicated genes or genomes such as soybean, it can avoid tedious and complicated procedure of crossing and screening through conventional breeding. GE technologies especially CRISPR based systems have evolved fast and most have been adopted to provide efficient tools for soybean improvement. The recent field trial of high oleic soybean using TALENs has demonstrated the bright future of soybean improvement if this technology is well implemented in plant breeding programs. At present, discovery of more GE target genes related agronomic important traits, adoption of newly developed GE technologies, simplification and renovation of editing reagent delivery and improvement of target mutant recovery method in soybean will expand editing outcomes, save time and reduce cost for product development. The cost-efficient preparation of intellectual property of GE technologies worked for soybean and understanding of GE related government regulation by breeders and farmers will promote GE product development. Transgene-free or DNA-free edited plants are considered as non-genetically modified events in several countries which will facilitate GE soybean production. Soybean is a commercial import and export crop with huge seed production. In the past, new technologies like transgenesis have been more widely and intensively applied to this crop compared to other crops. The recent advances in genome editing in soybean can potentially make it a leader once more in the era of new development in crop biotechnology.

## Author Contributions

YR wrote the manuscript. HX and LZ collected the materials. HX and KZ draw the figures. All authors contributed to the article and approved the submitted version.

## Conflict of Interest

HX, LZ, KZ, and YR were employed by company Tianjin Genovo Biotechnology, Co., Ltd. The authors declare that the research was conducted in the absence of any commercial or financial relationships that could be construed as a potential conflict of interest.

## Footnotes

1 http://www.fao.org/faostat/en/#data/QC

2 https://soybase.org/

3 http://bigd.big.ac.cn/gwh

4 https://calyxt.com/news/page/2/

5 http://cbi.hzau.edu.cn/crispr/

## References

[B1] Abudayyeh O. O. Gootenberg J. S. Essletzbichler P. Han S. Joung J. Belanto J. J. (2017). RNA targeting with CRISPR-Cas13. *Nature* 550 280–284. 10.1038/nature24049 28976959 PMC5706658

[B2] Abudayyeh O. O. Gootenberg J. S. Konermann S. Joung J. Slaymaker I. M. Cox D. B. (2016). C2c2 is a single-component programmable RNA-guided RNA-targeting CRISPR effector. *Science* 353:aaf5573. 10.1126/science.aaf5573 27256883 PMC5127784

[B3] Akond M. Liu S. Schoener L. Anderson J. A. Kantartzi S. K. Meksem K. (2013). A SNP-based genetic linkage map of soybean using the SoySNP6K illumina infinium beadchip genotyping array. *J. Plant Genome Sci.* 1 80–89. 10.5147/pggb.v1i3.154

[B4] al Amin N. Ahmad N. Wu N. Pu X. Ma T. Du Y. (2019). CRISPR-Cas9 mediated targeted disruption of FAD2-2 microsomal omega-6 desaturase in soybean (*Glycine max.* L). *BMC Biotechnol.* 19 1–10. 10.1186/s12896-019-0501-2 30691438 PMC6350355

[B5] Alok A. Kumar J. Upadhyay S. K. (2018). *Engineering in Hairy Roots using CRISPR/Cas9-Mediated Editing.* Singapore: Springer.

[B6] Aman R. Ali Z. Butt H. Mahas A. Aljedaani F. Khan M. Z. (2018). RNA virus interference via CRISPR/Cas13a system in plants. *Genome Biol.* 19:1. 10.1186/s13059-017-1381-1 29301551 PMC5755456

[B7] Andersson M. Turesson H. Olsson N. Falt A. S. Ohlsson P. Gonzalez M. N. (2018). Genome editing in potato *via* CRISPR-Cas9 ribonucleoprotein delivery. *Physiol. Plant* 164 378–384. 10.1111/ppl.12731 29572864

[B8] Anguraj Vadivel A. K. Krysiak K. Tian G. Dhaubhadel S. (2018). Genome-wide identification and localization of chalcone synthase family in soybean (*Glycine max* [L]Merr). *BMC Plant Biol.* 18:325. 10.1186/s12870-018-1569-x 30509179 PMC6278125

[B9] Anzalone A. V. Randolph P. B. Davis J. R. Sousa A. A. Koblan L. W. Levy J. M. (2019). Search-and-replace genome editing without double-strand breaks or donor DNA. *Nature* 576 149–157. 10.1038/s41586-019-1711-4 31634902 PMC6907074

[B10] Arikit S. Xia R. Kakrana A. Huang K. Zhai J. Yan Z. (2014). An atlas of soybean small RNAs identifies phased siRNAs from hundreds of coding genes. *Plant Cell* 26 4584–4601. 10.1105/tpc.114.131847 25465409 PMC4311202

[B11] Asaf S. Khan A. L. Al-Harrasi A. Kim T. H. Lee I.-J. (2018). The first complete mitochondrial genome of wild soybean (*Glycine soja*). *Mitochondr. DNA Part B* 3 527–528. 10.1080/23802359.2018.1467228 PMC7800914 33474228

[B12] Bae S. Park J. Kim J. S. (2014). Cas-OFFinder: a fast and versatile algorithm that searches for potential off-target sites of Cas9 RNA-guided endonucleases. *Bioinformatics* 30 1473–1475. 10.1093/bioinformatics/btu048 24463181 PMC4016707

[B13] Bai M. Yuan J. Kuang H. Gong P. Li S. Zhang Z. (2020). Generation of a multiplex mutagenesis population via pooled CRISPR-Cas9 in soya bean. *Plant Biotechnol. J.* 18 721–731. 10.1111/pbi.13239 31452351 PMC7004907

[B14] Bao A. Chen H. Chen L. Chen S. Hao Q. Guo W. (2019). CRISPR/Cas9-mediated targeted mutagenesis of *GmSPL9* genes alters plant architecture in soybean. *BMC Plant Biol.* 19:131. 10.1186/s12870-019-1746-6 30961525 PMC6454688

[B15] Begemann M. B. Gray B. N. January E. Singer A. Kesler D. C. He Y. (2017). Characterization and validation of a novel group of type V, class 2 nucleases for *in vivo* genome editing. *bioRxiv* 10.1101/192799

[B16] Beumer K. J. Trautman J. K. Christian M. Dahlem T. J. Lake C. M. Hawley R. S. (2013). Comparing zinc finger nucleases and transcription activator-like effector nucleases for gene targeting in Drosophila. *G3* 3 1717–1725. 10.1534/g3.113.007260 23979928 PMC3789796

[B17] Biden S. Smyth S. J. David Hudson D. (2018). The economic and environmental cost of delayed GM crop adoption: the case of Australia’s GM canola moratorium. *GM Crops Food* 9 13–20. 10.1080/21645698.2018.1429876 29359993 PMC5927647

[B18] Bonawitz N. D. Ainley W. M. Itaya A. Chennareddy S. R. Cicak T. Effinger K. (2019). Zinc finger nuclease-mediated targeting of multiple transgenes to an endogenous soybean genomic locus via non-homologous end joining. *Plant Biotechnol. J.* 17 750–761. 10.1111/pbi.13012 30220095 PMC6419576

[B19] Bortesi L. Fischer R. (2015). The CRISPR/Cas9 system for plant genome editing and beyond. *Biotechnol. Adv.* 33 41–52. 10.1016/j.biotechadv.2014.12.006 25536441

[B20] Brinegar K. Yetisen K. A. Choi S. Vallillo E. Ruiz-Esparza G. U. Prabhakar A. M. (2017). The commercialization of genome-editing technologies. *Crit. Rev. Biotechnol.* 37 924–932. 10.1080/07388551.2016.1271768 28100080

[B21] Brooks C. Nekrasov V. Lippman Z. B. Van Eck J. (2014). Efficient gene editing in tomato in the first generation using the clustered regularly interspaced short palindromic repeats/CRISPR-associated9 system. *Plant Physiol.* 166 1292–1297. 10.1104/pp.114.247577 25225186 PMC4226363

[B22] Burstein D. Harrington L. B. Strutt S. C. Probst A. J. Anantharaman K. Thomas B. C. (2017). New CRISPR-Cas systems from uncultivated microbes. *Nature* 542 237–241. 10.1038/nature21059 28005056 PMC5300952

[B23] Cai Y. Chen L. Liu X. Guo C. Sun S. Wu C. (2018). CRISPR/Cas9-mediated targeted mutagenesis of *GmFT2a* delays flowering time in soya bean. *Plant Biotechnol. J.* 16 176–185. 10.1111/pbi.12758 28509421 PMC5785355

[B24] Cai Y. Chen L. Liu X. Sun S. Wu C. Jiang B. (2015). CRISPR/Cas9-mediated genome editing in soybean hairy roots. *PLoS One* 10:e0136064. 10.1371/journal.pone.0136064 26284791 PMC4540462

[B25] Cai Y. Chen L. Zhang Y. Yuan S. Su Q. Sun S. (2020a). Target base editing in soybean using a modified CRISPR/Cas9 system. *Plant Biotechnol. J.* 10.1111/pbi.13386 [Epub ahead of print]. 32311214 PMC7540304

[B26] Cai Y. Wang L. Chen L. Wu T. Liu L. Sun S. (2020b). Mutagenesis of *GmFT2a* and *GmFT5a* mediated by CRISPR/Cas9 contributes for expanding the regional adaptability of soybean. *Plant Biotechnol. J* 18 298–309. 10.1111/pbi.13199 31240772 PMC6920152

[B27] Campbell B. W. Hoyle J. W. Bucciarelli B. Stec A. O. Samac D. A. Parrott W. A. (2019). Functional analysis and development of a CRISPR/Cas9 allelic series for a CPR5 ortholog necessary for proper growth of soybean trichomes. *Sci. Rep.* 9:14757. 10.1038/s41598-019-51240-7 31611562 PMC6791840

[B28] Cermak T. Doyle E. L. Christian M. Wang L. Zhang Y. Schmidt C. (2011). Efficient design and assembly of custom TALEN and other TAL effector-based constructs for DNA targeting. *Nucleic Acids Res.* 39:e82. 10.1093/nar/gkr218 21493687 PMC3130291

[B29] Chen F. Yang Y. Luo X. Zhou W. Dai Y. Zheng C. (2019). Genome-wide identification of GRF transcription factors in soybean and expression analysis of *GmGRF* family under shade stress. *BMC Plant Biol.* 19:269. 10.1186/s12870-019-1861-4 31226949 PMC6588917

[B30] Chen K. Wang Y. Zhang R. Zhang H. Gao C. (2019). CRISPR/Cas genome editing and precision plant breeding in agriculture. *Annu. Rev. Plant Biol.* 70 667–697. 10.1146/annurev-arplant-050718-100049 30835493

[B31] Chen L. Cai Y. Qu M. Wang L. Sun H. Jiang B. (2020a). Soybean adaption to high-latitude regions is associated with natural variations of *GmFT2b*, an ortholog of FLOWERING LOCUS T. *Plant Cell Environ.* 43 934–944. 10.1111/pce.13695 31981430 PMC7154755

[B32] Chen L. Nan H. Kong L. Yue L. Yang H. Zhao Q. (2020b). Soybean AP1 homologs control flowering time and plant height. *J. Integr. Plant Biol.* [Epub ahead of print] 10.1111/jipb.12988 32619080

[B33] Chen W. Song K. Cai Y. Li W. Liu B. Liu L. (2011). Genetic modification of soybean with a novel grafting technique: downregulating the *FAD2-1* gene increases oleic acid content. *Plant Mol. Biol. Rep.* 29 866–874. 10.1007/s11105-011-0286-5

[B34] Cheng Q. Dong L. Su T. Li T. Gan Z. Nan H. (2019). CRISPR/Cas9-mediated targeted mutagenesis of *GmLHY* genes alters plant height and internode length in soybean. *BMC Plant Biol.* 19:562. 10.1186/s12870-019-2145-8 31852439 PMC6921449

[B35] Choi I. Y. Hyten D. L. Matukumalli L. K. Song Q. Chaky J. M. Quigley C. V. (2007). A soybean transcript map: gene distribution, haplotype and single-nucleotide polymorphism analysis. *Genetics* 176 685–696. 10.1534/genetics.107.070821 17339218 PMC1893076

[B36] Chung G. Singh R. J. (2008). Broadening the genetic base of soybean: a multidisciplinary approach. *Crit. Rev. Plant Sci.* 27 295–341. 10.1080/07352680802333904

[B37] Chung W. H. Jeong N. Kim J. Lee W. K. Lee Y. G. Lee S. H. (2014). Population structure and domestication revealed by high-depth resequencing of Korean cultivated and wild soybean genomes. *DNA Res.* 21 153–167. 10.1093/dnares/dst047 24271940 PMC3989487

[B38] Cong L. Ran F. A. Cox D. Lin S. Barretto R. Habib N. (2013). Multiplex genome engineering using CRISPR/Cas systems. *Science* 339 819–823. 10.1126/science.1231143 23287718 PMC3795411

[B39] Corbesier L. Coupland G. (2006). The quest for florigen: a review of recent progress. *J. Exp. Bot.* 57 3395–3403. 10.1093/jxb/erl095 17030536

[B40] Cox D. B. T. Gootenberg J. S. Abudayyeh O. O. Franklin B. Kellner M. J. Joung J. (2017). RNA editing with CRISPR-Cas13. *Science* 358 1019–1027. 10.1126/science.aaq0180 29070703 PMC5793859

[B41] Curtin S. J. Xiong Y. Michno J. M. Campbell B. W. Stec A. O. Cermak T. (2018). CRISPR/Cas9 and TALENs generate heritable mutations for genes involved in small RNA processing of *Glycine max* and *Medicago truncatula*. *Plant Biotechnol. J.* 16 1125–1137. 10.1111/pbi.12857 29087011 PMC5978873

[B42] Curtin S. J. Zhang F. Sander J. D. Haun W. J. Starker C. Baltes N. J. (2011). Targeted mutagenesis of duplicated genes in soybean with zinc-finger nucleases. *Plant Physiol.* 156 466–473. 10.1104/pp.111.172981 21464476 PMC3177250

[B43] Debernardi J. M. Tricoli D. M. Ercoli M. F. Hayta S. Ronald P. Palatnik J. F. (2020). A chimera including a GROWTH-REGULATING FACTOR (GRF) and its cofactor GRF-INTERACTING FACTOR (GIF) increases transgenic plant regeneration efficiency. *bioRxiv* 10.1101/2020.08.23.263905

[B44] Demorest Z. L. Coffman A. Baltes N. J. Stoddard T. J. Clasen B. M. Luo S. (2016). Direct stacking of sequence-specific nuclease-induced mutations to produce high oleic and low linolenic soybean oil. *BMC Plant Biol.* 16:225. 10.1186/s12870-016-0906-1 27733139 PMC5062912

[B45] Di Y. H. Sun X. J. Hu Z. Jiang Q. Y. Song G. H. Zhang B. (2019). Enhancing the CRISPR/Cas9 system based on multiple GmU6 promoters in soybean. *Biochem. Biophys. Res. Commun.* 519 819–823. 10.1016/j.bbrc.2019.09.074 31558318

[B46] Do P. T. Nguyen C. X. Bui H. T. Tran L. T. N. Stacey G. Gillman J. D. (2019). Demonstration of highly efficient dual gRNA CRISPR/Cas9 editing of the homeologous *GmFAD2-1A* and *GmFAD2-1B* genes to yield a high oleic, low linoleic and alpha-linolenic acid phenotype in soybean. *BMC Plant Biol.* 19:311. 10.1186/s12870-019-1906-8 31307375 PMC6632005

[B47] Domitrovich A. M. Kunkel G. R. (2003). Multiple, dispersed human U6 small nuclear RNA genes with varied transcriptional efficiencies. *Nucleic Acids Res.* 31 2344–2352. 10.1093/nar/gkg331 12711679 PMC154217

[B48] Doudna J. A. Charpentier E. (2014). Genome editing. The new frontier of genome engineering with CRISPR-Cas9. *Science* 346:1258096. 10.1126/science.1258096 25430774

[B49] Du H. Zeng X. Zhao M. Cui X. Wang Q. Yang H. (2016). Efficient targeted mutagenesis in soybean by TALENs and CRISPR/Cas9. *J. Biotechnol.* 217 90–97. 10.1016/j.jbiotec.2015.11.005 26603121

[B50] Friedrichs S. Takasu Y. Kearns P. Dagallier B. Oshima R. Schofield J. (2019). An overview of regulatory approaches to genome editing in agriculture. *Biotechnol. Res. Innov.* 3 208–220. 10.1016/j.biori.2019.07.001

[B51] Gale F. Valdes C. Ash M. (2019). *Interdependence of China, United States, and Brazil in Soybean Trade.* New York, NY: US Department of Agriculture’s Economic Research Service.

[B52] Gao C.-W. Gao L.-Z. (2017). The complete chloroplast genome sequence of semi-wild soybean, *Glycine gracilis* (Fabales: Fabaceae). *Conserv. Genet. Resour.* 9 343–345. 10.1007/s12686-016-0683-z

[B53] Gao L. Cox D. B. T. Yan W. X. Manteiga J. C. Schneider M. W. Yamano T. (2017). Engineered Cpf1 variants with altered PAM specificities. *Nat. Biotechnol.* 35 789–792. 10.1038/nbt.3900 28581492 PMC5548640

[B54] Gaudelli N. M. Komor A. C. Rees H. A. Packer M. S. Badran A. H. Bryson D. I. (2017). Programmable base editing of A^∗^T to G^∗^C in genomic DNA without DNA cleavage. *Nature* 551 464–471. 10.1038/nature24644 29160308 PMC5726555

[B55] Gilbert L. A. Larson M. H. Morsut L. Liu Z. Brar G. A. Torres S. E. (2013). CRISPR-mediated modular RNA-guided regulation of transcription in eukaryotes. *Cell* 154 442–451. 10.1016/j.cell.2013.06.044 23849981 PMC3770145

[B56] Hada A. Krishnan V. Jaabir M. S. M. Kumari A. Jolly M. Praveen S. (2018). Improved *Agrobacterium tumefaciens*-mediated transformation of soybean [*Glycine max* (L.) Merr.] following optimization of culture conditions and mechanical techniques. *In Vitro Cell. Dev. Biol. Plant* 54 672–688. 10.3390/ijms19103039 30301169 PMC6213721

[B57] Haeussler M. Schonig K. Eckert H. Eschstruth A. Mianne J. Renaud J. B. (2016). Evaluation of off-target and on-target scoring algorithms and integration into the guide RNA selection tool CRISPOR. *Genome Biol.* 17:148. 10.1186/s13059-016-1012-2 27380939 PMC4934014

[B58] Hahn F. Nekrasov V. (2019). CRISPR/Cas precision: do we need to worry about off-targeting in plants? *Plant Cell Rep.* 38 437–441. 10.1007/s00299-018-2355-9 30426198 PMC6469637

[B59] Han J. Guo B. Guo Y. Zhang B. Wang X. Qiu L. J. (2019). Creation of early flowering germplasm of soybean by CRISPR/Cas9 technology. *Front. Plant Sci.* 10:1446. 10.3389/fpls.2019.01446 31824524 PMC6882952

[B60] Hart C. (2017). “The economic evolution of the soybean industry,” in *The Soybean Genome*, eds Nguyen H. Bhattacharyya M. (Cham: Springer), 1–9. 10.1007/978-3-319-64198-0_1

[B61] Haun W. Coffman A. Clasen B. M. Demorest Z. L. Lowy A. Ray E. (2014). Improved soybean oil quality by targeted mutagenesis of the fatty acid desaturase 2 gene family. *Plant Biotechnol. J.* 12 934–940. 10.1111/pbi.12201 24851712

[B62] Homrich M. S. Wiebke-Strohm B. Weber R. L. Bodanese-Zanettini M. H. (2012). Soybean genetic transformation: a valuable tool for the functional study of genes and the production of agronomically improved plants. *Genet. Mol. Biol.* 35(Suppl. 4) 998–1010. 10.1590/s1415-47572012000600015 23412849 PMC3571417

[B63] Jacobs T. B. LaFayette P. R. Schmitz R. J. Parrott W. A. (2015). Targeted genome modifications in soybean with CRISPR/Cas9. *BMC Biotechnol.* 15:16. 10.1186/s12896-015-0131-2 25879861 PMC4365529

[B64] Ji X. Yang B. Wang D. (2020). Achieving plant genome editing while bypassing tissue culture. *Trends Plant Sci.* 25 427–429. 10.1016/j.tplants.2020.02.011 32304655

[B65] Jiang W. Zhou H. Bi H. Fromm M. Yang B. Weeks D. P. (2013). Demonstration of CRISPR/Cas9/sgRNA-mediated targeted gene modification in Arabidopsis, tobacco, sorghum and rice. *Nucleic Acids Res.* 41:e188. 10.1093/nar/gkt780 23999092 PMC3814374

[B66] Jinek M. Chylinski K. Fonfara I. Hauer M. Doudna J. A. Charpentier E. (2012). A programmable dual-RNA-guided DNA endonuclease in adaptive bacterial immunity. *Science* 337 816–821. 10.1126/science.1225829 22745249 PMC6286148

[B67] Joung J. K. Sander J. D. (2013). TALENs: a widely applicable technology for targeted genome editing. *Nat. Rev. Mol. Cell Biol.* 14 49–55. 10.1038/nrm3486 23169466 PMC3547402

[B68] Kanazashi Y. Hirose A. Takahashi I. Mikami M. Endo M. Hirose S. (2018). Simultaneous site-directed mutagenesis of duplicated loci in soybean using a single guide RNA. *Plant Cell Rep.* 37 553–563. 10.1007/s00299-018-2251-3 29333573

[B69] Kardailsky I. Shukla V. K. Ahn J. H. Dagenais N. Christensen S. K. Nguyen J. T. (1999). Activation tagging of the floral inducer FT. *Science* 286 1962–1965. 10.1126/science.286.5446.1962 10583961

[B70] Karpechenko G. D. (1925). On the chromosomes of Phaseolinae. *Bull. Appl. Bot. Genet. Breed.* 14 143–148.

[B71] Kim H. Kim S. T. Ryu J. Kang B. C. Kim J. S. Kim S. G. (2017). CRISPR/Cpf1-mediated DNA-free plant genome editing. *Nat. Commun.* 8:14406. 10.1038/ncomms14406 28205546 PMC5316869

[B72] Kim M. Y. Lee S. Van K. Kim T. H. Jeong S. C. Choi I. Y. (2010). Whole-genome sequencing and intensive analysis of the undomesticated soybean *(Glycine soja* Sieb. and Zucc.) genome. *Proc. Natl. Acad. Sci. U.S.A.* 107 22032–22037. 10.1073/pnas.1009526107 21131573 PMC3009785

[B73] Komor A. C. Kim Y. B. Packer M. S. Zuris J. A. Liu D. R. (2016). Programmable editing of a target base in genomic DNA without double-stranded DNA cleavage. *Nature* 533 420–424. 10.1038/nature17946 27096365 PMC4873371

[B74] Komor A. C. Zhao K. T. Packer M. S. Gaudelli N. M. Waterbury A. L. Koblan L. W. (2017). Improved base excision repair inhibition and bacteriophage Mu Gam protein yields C:G-to-T:A base editors with higher efficiency and product purity. *Sci. Adv.* 3:eaao4774. 10.1126/sciadv.aao4774 28875174 PMC5576876

[B75] Kong F. Liu B. Xia Z. Sato S. Kim B. M. Watanabe S. (2010). Two coordinately regulated homologs of FLOWERING LOCUS T are involved in the control of photoperiodic flowering in soybean. *Plant Physiol.* 154 1220–1231. 10.1104/pp.110.160796 20864544 PMC2971601

[B76] Koonin E. V. Makarova K. S. Zhang F. (2017). Diversity, classification and evolution of CRISPR-Cas systems. *Curr. Opin. Microbiol.* 37 67–78. 10.1016/j.mib.2017.05.008 28605718 PMC5776717

[B77] Lakhssassi N. Liu S. Bekal S. Zhou Z. Colantonio V. Lambert K. (2017). Characterization of the soluble NSF attachment protein gene family identifies two members involved in additive resistance to a plant pathogen. *Sci. Rep.* 7:45226. 10.1038/srep45226 28338077 PMC5364553

[B78] Lam H. M. Xu X. Liu X. Chen W. Yang G. Wong F. L. (2010). Resequencing of 31 wild and cultivated soybean genomes identifies patterns of genetic diversity and selection. *Nat. Genet.* 42 1053–1059. 10.1038/ng.715 21076406

[B79] Li B. Fillmore N. Bai Y. Collins M. Thomson J. A. Stewart R. (2014). Evaluation of de novo transcriptome assemblies from RNA-Seq data. *Genome Biol.* 15:553. 10.1186/s13059-014-0553-5 25608678 PMC4298084

[B80] Li Y. H. Zhou G. Ma J. Jiang W. Jin L. G. Zhang Z. (2014). De novo assembly of soybean wild relatives for pan-genome analysis of diversity and agronomic traits. *Nat Biotechnol* 32 1045–1052. 10.1038/nbt.2979 25218520

[B81] Li C. Nguyen V. Liu J. Fu W. Chen C. Yu K. (2019). Mutagenesis of seed storage protein genes in soybean using CRISPR/Cas9. *BMC Res. Notes* 12:176. 10.1186/s13104-019-4207-2 30917862 PMC6437971

[B82] Li R. Qiu Z. Wang X. Gong P. Xu Q. Yu Q. B. (2019). Pooled CRISPR/Cas9 reveals redundant roles of plastidial phosphoglycerate kinases in carbon fixation and metabolism. *Plant J.* 98 1078–1089. 10.1111/tpj.14303 30834637

[B83] Li H. Li J. Chen J. Yan L. Xia L. (2020). Precise modifications of both exogenous and endogenous genes in rice by prime editing. *Mol. Plant* 13 671–674. 10.1016/j.molp.2020.03.011 32222486

[B84] Li J. F. Norville J. E. Aach J. McCormack M. Zhang D. Bush J. (2013). Multiplex and homologous recombination-mediated genome editing in Arabidopsis and *Nicotiana benthamiana* using guide RNA and Cas9. *Nat. Biotechnol.* 31 688–691. 10.1038/nbt.2654 23929339 PMC4078740

[B85] Li S. Cong Y. Liu Y. Wang T. Shuai Q. Chen N. (2017). Optimization of *Agrobacterium*-mediated transformation in soybean. *Front. Plant Sci.* 8:246. 10.3389/fpls.2017.00246 28286512 PMC5323423

[B86] Li S. Zhang X. Wang W. Guo X. Wu Z. Du W. (2018). Expanding the scope of CRISPR/Cpf1-mediated genome editing in rice. *Mol. Plant* 11 995–998. 10.1016/j.molp.2018.03.009 29567453

[B87] Li Z. Liu Z. B. Xing A. Moon B. P. Koellhoffer J. P. Huang L. (2015). Cas9-guide RNA directed genome editing in soybean. *Plant Physiol.* 169 960–970. 10.1104/pp.15.00783 26294043 PMC4587461

[B88] Liang Z. Chen K. Li T. Zhang Y. Wang Y. Zhao Q. (2017). Efficient DNA-free genome editing of bread wheat using CRISPR/Cas9 ribonucleoprotein complexes. *Nat. Commun.* 8:14261. 10.1038/ncomms14261 28098143 PMC5253684

[B89] Lin Q. Zong Y. Xue C. Wang S. Jin S. Zhu Z. (2020). Prime genome editing in rice and wheat. *Nat. Biotechnol.* 38 582–585. 10.1038/s41587-020-0455-x 32393904

[B90] Liu J. Gunapati S. Mihelich N. T. Stec A. O. Michno J. M. Stupar R. M. (2019). Genome editing in soybean with CRISPR/Cas9. *Methods Mol. Biol.* 1917 217–234. 10.1007/978-1-4939-8991-1_16 30610639

[B91] Liu J. J. Orlova N. Oakes B. L. Ma E. Spinner H. B. Baney K. L. M. (2019). CasX enzymes comprise a distinct family of RNA-guided genome editors. *Nature* 566 218–223. 10.1038/s41586-019-0908-x 30718774 PMC6662743

[B92] Liu S. NaoufalLakhssassi, Zhou Z. Colantonio V. Kassem M. A. Meksem K. (2017). “Soybean genomic libraries, TILLING, and genetic resources,” in *The Soybean Genome*, eds Nguyen H. Bhattacharyya M. (Cham: Springer).

[B93] Lowe K. Wu E. Wang N. Hoerster G. Hastings C. Cho M. J. (2016). Morphogenic regulators Baby boom and Wuschel improve monocot transformation. *Plant Cell* 28 1998–2015. 10.1105/tpc.16.00124 27600536 PMC5059793

[B94] Makarova K. S. Wolf Y. I. Alkhnbashi O. S. Costa F. Shah S. A. Saunders S. J. (2015). An updated evolutionary classification of CRISPR-Cas systems. *Nat. Rev. Microbiol.* 13 722–736. 10.1038/nrmicro3569 26411297 PMC5426118

[B95] Mali P. Aach J. Stranges P. B. Esvelt K. M. Moosburner M. Kosuri S. (2013). CAS9 transcriptional activators for target specificity screening and paired nickases for cooperative genome engineering. *Nat. Biotechnol.* 31 833–838. 10.1038/nbt.2675 23907171 PMC3818127

[B96] Mangena P. (2019). A simplified in-planta genetic transformation in soybean. *Res. J. Biotechnol.* 14 117–125.

[B97] Mao Y. Zhang Z. Feng Z. Wei P. Zhang H. Botella J. R. (2016). Development of germ-line-specific CRISPR-Cas9 systems to improve the production of heritable gene modifications in Arabidopsis. *Plant Biotechnol. J.* 14 519–532. 10.1111/pbi.12468 26360626 PMC5515382

[B98] Metje-Sprink J. Sprink T. Hartung F. (2020). Genome-edited plants in the field. *Curr. Opin. Biotechnol.* 61 1–6. 10.1016/j.copbio.2019.08.007 31557656

[B99] Michno J. M. Virdi K. Stec A. O. Liu J. Wang X. Xiong Y. (2020). Integration, abundance, and transmission of mutations and transgenes in a series of CRISPR/Cas9 soybean lines. *BMC Biotechnol.* 20:10. 10.1186/s12896-020-00604-3 32093670 PMC7038615

[B100] Michno J. M. Wang X. Liu J. Curtin S. J. Kono T. J. Stupar R. M. (2015). CRISPR/Cas mutagenesis of soybean and *Medicago truncatula* using a new web-tool and a modified Cas9 enzyme. *GM Crops Food* 6 243–252. 10.1080/21645698.2015.1106063 26479970 PMC5033229

[B101] Mishra R. Joshi R. K. Zhao K. (2020). Base editing in crops: current advances, limitations and future implications. *Plant Biotechnol. J.* 18 20–31. 10.1111/pbi.13225 31365173 PMC6920333

[B102] Mohammed S. Abd Samad A. Rahmat Z. (2019). *Agrobacterium*-mediated transformation of rice: constraints and possible solutions. *Rice Sci.* 26 133–146. 10.1016/j.rsci.2019.04.001

[B103] Mookkan M. Nelson-Vasilchik K. Hague J. Zhang Z. J. Kausch A. P. (2017). Selectable marker independent transformation of recalcitrant maize inbred B73 and sorghum P898012 mediated by morphogenic regulators BABY BOOM and WUSCHEL2. *Plant Cell Rep.* 36 1477–1491. 10.1007/s00299-017-2169-1 28681159 PMC5565672

[B104] Murugan K. Babu K. Sundaresan R. Rajan R. Sashital D. G. (2017). The revolution continues: newly discovered systems expand the CRISPR-Cas toolkit. *Mol. Cell* 68 15–25. 10.1016/j.molcel.2017.09.007 28985502 PMC5683099

[B105] Nekrasov V. Staskawicz B. Weigel D. Jones J. D. Kamoun S. (2013). Targeted mutagenesis in the model plant *Nicotiana benthamiana* using Cas9 RNA-guided endonuclease. *Nat. Biotechnol.* 31 691–693. 10.1038/nbt.2655 23929340

[B106] O’Connell M. R. Oakes B. L. Sternberg S. H. East-Seletsky A. Kaplan M. Doudna J. A. (2014). Programmable RNA recognition and cleavage by CRISPR/Cas9. *Nature* 516 263–266. 10.1038/nature13769 25274302 PMC4268322

[B107] O’Rourke J. A. Graham M. A. Whitham S. A. (2017). “Soybean functional genomics: bridging the genotype-to-phenotype gap,” in *The Soybean Genome. Compendium of Plant Genomes*, eds Nguyen H. Bhattacharyya M. (Cham: Springer).

[B108] Petolino J. F. (2015). Genome editing in plants via designed zinc finger nucleases. *In Vitro Cell Dev. Biol. Plant* 51 1–8. 10.1007/s11627-015-9663-3 25774080 PMC4352198

[B109] Ran Y. Liang Z. Gao C. (2017). Current and future editing reagent delivery systems for plant genome editing. *Sci. China Life Sci.* 60 490–505. 10.1007/s11427-017-9022-1 28527114

[B110] Rech E. L. Vianna G. R. Aragao F. J. (2008). High-efficiency transformation by biolistics of soybean, common bean and cotton transgenic plants. *Nat. Protoc.* 3 410–418. 10.1038/nprot.2008.9 18323812

[B111] Sander J. D. Dahlborg E. J. Goodwin M. J. Cade L. Zhang F. Cifuentes D. (2011). Selection-free zinc-finger-nuclease engineering by context-dependent assembly (CoDA). *Nat. Methods* 8 67–69. 10.1038/nmeth.1542 21151135 PMC3018472

[B112] Scheben A. Edwards D. (2018). Bottlenecks for genome-edited crops on the road from lab to farm. *Genome Biol.* 19:178. 10.1186/s13059-018-1555-5 30367679 PMC6202801

[B113] Schmidt S. M. Belisle M. Frommer W. B. (2020). The evolving landscape around genome editing in agriculture: many countries have exempted or move to exempt forms of genome editing from GMO regulation of crop plants. *Embo Rep.* 21:e50680. 10.15252/embr.202050680 PMC7271327 32431018

[B114] Schmutz J. Cannon S. B. Schlueter J. Ma J. Mitros T. Nelson W. (2010). Genome sequence of the palaeopolyploid soybean. *Nature* 463 178–183. 10.1038/nature08670 20075913

[B115] Sen N. K. Vidyabhusan R. V. (1960). Tetraploid soybeans. *Euphytica* 9 317–322.

[B116] Shan Q. Wang Y. Li J. Gao C. (2014). Genome editing in rice and wheat using the CRISPR/Cas system. *Nat. Protoc.* 9 2395–2410. 10.1038/nprot.2014.157 25232936

[B117] Shan Q. Wang Y. Li J. Zhang Y. Chen K. Liang Z. (2013). Targeted genome modification of crop plants using a CRISPR-Cas system. *Nat. Biotechnol.* 31 686–688. 10.1038/nbt.2650 23929338

[B118] Shen B. Zhang W. Zhang J. Zhou J. Wang J. Chen L. (2014). Efficient genome modification by CRISPR-Cas9 nickase with minimal off-target effects. *Nat. Methods* 11 399–402. 10.1038/nmeth.2857 24584192

[B119] Shen Y. Liu J. Geng H. Zhang J. Liu Y. Zhang H. (2018). De novo assembly of a Chinese soybean genome. *Sci. China Life Sci.* 61 871–884. 10.1007/s11427-018-9360-0 30062469

[B120] Shoemaker R. C. Schlueter J. Doyle J. J. (2006). Paleopolyploidy and gene duplication in soybean and other legumes. *Curr. Opin. Plant Biol.* 9 104–109. 10.1016/j.pbi.2006.01.007 16458041

[B121] Singh R. Kuscu C. Quinlan A. Qi Y. Adli M. (2015). Cas9-chromatin binding information enables more accurate CRISPR off-target prediction. *Nucleic Acids Res.* 43:e118. 10.1093/nar/gkv575 26032770 PMC4605288

[B122] Singh R. J. (2017). “Botany and cytogenetics of soybean,” in *The Soybean Genome. Compendium of Plant Genomes*, eds Nguyen H. Bhattacharyya M. (Cham: Springer).

[B123] Sprink T. Eriksson D. Schiemann J. Hartung F. (2016). Regulatory hurdles for genome editing: process- vs. product-based approaches in different regulatory contexts. *Plant Cell Rep.* 35 1493–1506. 10.1007/s00299-016-1990-2 27142995 PMC4903111

[B124] Sun X. Hu Z. Chen R. Jiang Q. Song G. Zhang H. (2015). Targeted mutagenesis in soybean using the CRISPR-Cas9 system. *Sci. Rep.* 5:10342. 10.1038/srep10342 26022141 PMC4448504

[B125] Svitashev S. Schwartz C. Lenderts B. Young J. K. Mark Cigan A. (2016). Genome editing in maize directed by CRISPR-Cas9 ribonucleoprotein complexes. *Nat. Commun.* 7:13274. 10.1038/ncomms13274 27848933 PMC5116081

[B126] Tang X. Sretenovic S. Ren Q. Jia X. Li M. Fan T. (2020). Plant prime editors enable precise gene editing in rice cells. *Mol. Plant* 13 667–670. 10.1016/j.molp.2020.03.010 32222487

[B127] Teng F. Cui T. Feng G. Guo L. Xu K. Gao Q. (2018). Repurposing CRISPR-Cas12b for mammalian genome engineering. *Cell Discov.* 4:63. 10.1038/s41421-018-0069-3 30510770 PMC6255809

[B128] Teng F. Li J. Cui T. Xu K. Guo L. Gao Q. (2019). Enhanced mammalian genome editing by new Cas12a orthologs with optimized crRNA scaffolds. *Genome Biol.* 20:15. 10.1186/s13059-019-1620-8 30717767 PMC6362571

[B129] Turck F. Fornara F. Coupland G. (2008). Regulation and identity of florigen: FLOWERING LOCUS T moves center stage. *Annu. Rev. Plant Biol.* 59 573–594. 10.1146/annurev.arplant.59.032607.092755 18444908

[B130] Voytas D. F. Gao C. (2014). Precision genome engineering and agriculture: opportunities and regulatory challenges. *PLoS Biol.* 12:e1001877. 10.1371/journal.pbio.1001877 24915127 PMC4051594

[B131] Wang J. Kuang H. Zhang Z. Yang Y. Guan Y. (2020). Generation of seed lipoxygenase-free soybean using CRISPR-Cas9. *Crop J.* 8 432–439. 10.1016/j.cj.2019.08.008

[B132] Wang L. Sun S. Wu T. Liu L. Sun X. Cai Y. (2020). Natural variation and CRISPR/Cas9-mediated mutation in GmPRR37 affect photoperiodic flowering and contribute to regional adaptation of soybean. *Plant Biotechnol. J.* 18 1869–1881. 10.1111/pbi.13346 31981443 PMC7415786

[B133] Wang M. B. Helliwell C. A. Wu L. M. Waterhouse P. M. Peacock W. J. Dennis E. S. (2008). Hairpin RNAs derived from RNA polymerase II and polymerase III promoter-directed transgenes are processed differently in plants. *RNA* 14 903–913. 10.1261/rna.760908 18367720 PMC2327362

[B134] Wang S. Yokosho K. Guo R. Whelan J. Ruan Y. L. Ma J. F. (2019). The soybean sugar transporter *GmSWEET15* mediates sucrose export from endosperm to early embryo. *Plant Physiol.* 180 2133–2141. 10.1104/pp.19.00641 31221732 PMC6670074

[B135] Wang Y. Yuan L. Su T. Wang Q. Gao Y. Zhang S. (2019). Light- and temperature-entrainable circadian clock in soybean development. *Plant Cell Environ.* 43 637–648. 10.1111/pce.13678 31724182

[B136] Wang Z. P. Xing H. L. Dong L. Zhang H. Y. Han C. Y. Wang X. C. (2015). Egg cell-specific promoter-controlled CRISPR/Cas9 efficiently generates homozygous mutants for multiple target genes in Arabidopsis in a single generation. *Genome Biol.* 16:144. 10.1186/s13059-015-0715-0 26193878 PMC4507317

[B137] Wang Z. Y. Tobin E. M. (1998). Constitutive expression of the CIRCADIAN CLOCK ASSOCIATED 1 (CCA1) gene disrupts circadian rhythms and suppresses its own expression. *Cell* 93 1207–1217. 10.1016/s0092-8674(00)81464-6 9657153

[B138] Weeks D. P. Spalding M. H. Yang B. (2016). Use of designer nucleases for targeted gene and genome editing in plants. *Plant Biotechnol. J.* 14 483–495. 10.1111/pbi.12448 26261084 PMC11388832

[B139] Woo J. W. Kim J. Kwon S. I. Corvalan C. Cho S. W. Kim H. (2015). DNA-free genome editing in plants with preassembled CRISPR-Cas9 ribonucleoproteins. *Nat. Biotechnol.* 33 1162–1164. 10.1038/nbt.3389 26479191

[B140] Wright D. A. Townsend J. A. Winfrey R. J. Jr. Irwin P. A. Rajagopal J. (2005). High-frequency homologous recombination in plants mediated by zinc-finger nucleases. *Plant J.* 44 693–705. 10.1111/j.1365-313X.2005.02551.x 16262717

[B141] Wu N. Lu Q. Wang P. Zhang Q. Zhang J. Qu J. (2020). Construction and analysis of *GmFAD2-1A* and *GmFAD2-2A* soybean fatty acid desaturase mutants based on CRISPR/Cas9 technology. *Int. J. Mol. Sci.* 21:1104. 10.3390/ijms21031104 32046096 PMC7037799

[B142] Xia Z. Tsubokura Y. Hoshi M. Hanawa M. Yano C. Okamura K. (2007). An integrated high-density linkage map of soybean with RFLP, SSR, STS, and AFLP markers using A single F2 population. *DNA Res.* 14 257–269. 10.1093/dnares/dsm027 18192280 PMC2779910

[B143] Xie M. Chung C. Y. Li M. W. Wong F. L. Wang X. Liu A. (2019). A reference-grade wild soybean genome. *Nat. Commun.* 10:1216. 10.1038/s41467-019-09142-9 30872580 PMC6418295

[B144] Xu Y. Meng X. Wang J. Qin B. Wang K. Li J. (2020). ScCas9 recognizes NNG protospacer adjacent motif in genome editing of rice. *Sci. China Life Sci.* 63 450–452. 10.1007/s11427-019-1630-2 31953707

[B145] Yamada T. Takagi K. Ishimoto M. (2012). Recent advances in soybean transformation and their application to molecular breeding and genomic analysis. *Breed Sci.* 61 480–494. 10.1270/jsbbs.61.480 23136488 PMC3406787

[B146] Yan L. Wei S. Wu Y. Hu R. Li H. Yang W. (2015). High-efficiency genome editing in Arabidopsis using YAO promoter-driven CRISPR/Cas9 system. *Mol. Plant* 8 1820–1823. 10.1016/j.molp.2015.10.004 26524930

[B147] Yang J. Xing G. Niu L. He H. Guo D. Du Q. (2018). Improved oil quality in transgenic soybean seeds by RNAi-mediated knockdown of *GmFAD2-1B*. *Transgenic Res.* 27 155–166. 10.1007/s11248-018-0063-4 29476327

[B148] Yang L. Yang B. Chen J. (2019). One prime for all editing. *Cell* 179 1448–1450. 10.1016/j.cell.2019.11.030 31835025

[B149] Yin G. Xu H. Xiao S. Qin Y. Li Y. Yan Y. (2013). The large soybean (*Glycine max*) WRKY TF family expanded by segmental duplication events and subsequent divergent selection among subgroups. *BMC Plant Biol.* 13:148. 10.1186/1471-2229-13-148 24088323 PMC3850935

[B150] Young J. Zastrow-Hayes G. Deschamps S. Svitashev S. Zaremba M. Acharya A. (2019). CRISPR-Cas9 Editing in maize: systematic evaluation of off-target activity and its relevance in crop improvement. *Sci. Rep.* 9:6729. 10.1038/s41598-019-43141-6 31040331 PMC6491584

[B151] Zetsche B. Gootenberg J. S. Abudayyeh O. O. Slaymaker I. M. Makarova K. S. Essletzbichler P. (2015). Cpf1 is a single RNA-guided endonuclease of a class 2 CRISPR-Cas system. *Cell* 163 759–771. 10.1016/j.cell.2015.09.038 26422227 PMC4638220

[B152] Zetsche B. Strecker J. Abudayyeh O. O. Gootenberg J. S. Scott D. A. Zhang F. (2017). A survey of genome editing activity for 16 Cpf1 orthologs. *bioRxiv* 10.1101/134015 PMC7220826 31723075

[B153] Zhang D. Zhang H. Li T. Chen K. Qiu J. L. Gao C. (2017). Perfectly matched 20-nucleotide guide RNA sequences enable robust genome editing using high-fidelity SpCas9 nucleases. *Genome Biol.* 18:191. 10.1186/s13059-017-1325-9 29020979 PMC5637269

[B154] Zhang T. Zhao Y. Ye J. Cao X. Xu C. Chen B. (2019). Establishing CRISPR/Cas13a immune system conferring RNA virus resistance in both dicot and monocot plants. *Plant Biotechnol. J.* 17 1185–1187. 10.1111/pbi.13095 30785668 PMC6576088

[B155] Zhang Y. Malzahn A. A. Sretenovic S. Qi Y. (2019). The emerging and uncultivated potential of CRISPR technology in plant science. *Nat. Plants* 5 778–794. 10.1038/s41477-019-0461-5 31308503

[B156] Zhang X. H. Tee L. Y. Wang X. G. Huang Q. S. Yang S. H. (2015). Off-target effects in CRISPR/Cas9-mediated genome engineering. *Mol. Ther. Nucleic Acids* 4:e264. 10.1038/mtna.2015.37 26575098 PMC4877446

[B157] Zheng N. Li T. Dittman J. D. Su J. Li R. Gassmann W. (2020). CRISPR/Cas9-based gene editing using egg cell-specific promoters in Arabidopsis and soybean. *Front. Plant Sci.* 11:800. 10.3389/fpls.2020.00800 32612620 PMC7309964

[B158] Zhong Z. Sretenovic S. Ren Q. Yang L. Bao Y. Qi C. (2019). Improving plant genome editing with high-fidelity xCas9 and non-canonical PAM-targeting Cas9-NG. *Mol. Plant* 12 1027–1036. 10.1016/j.molp.2019.03.011 30928637

[B159] Zhong Z. Zhang Y. You Q. Tang X. Ren Q. Liu S. (2018). Plant genome editing using *FnCpf1* and *LbCpf1* nucleases at redefined and altered PAM sites. *Mol. Plant* 11 999–1002. 10.1016/j.molp.2018.03.008 29567452

[B160] Zhou Z. Jiang Y. Wang Z. Gou Z. Lyu J. Li W. (2015). Resequencing 302 wild and cultivated accessions identifies genes related to domestication and improvement in soybean. *Nat. Biotechnol.* 33 408–414. 10.1038/nbt.3096 25643055

[B161] Zhu Y. Wu N. Song W. Yin G. Qin Y. Yan Y. (2014). Soybean (*Glycine max*) expansin gene superfamily origins: segmental and tandem duplication events followed by divergent selection among subfamilies. *BMC Plant Biol.* 14:93. 10.1186/1471-2229-14-93 24720629 PMC4021193

[B162] Zou P. Duan L. Zhang S. Bai X. Liu Z. Jin F. (2020). Target specificity of the CRISPR-Cas9 system in *Arabidopsis thaliana*, *Oryza sativa*, and *Glycine max* genomes. *J. Comput. Biol.* [Epub ahead of print] 10.1089/cmb.2019.0453 32298599

